# Information and Communication Technology–Enabled Person-Centered Care for the “Big Five” Chronic Conditions: Scoping Review

**DOI:** 10.2196/jmir.3687

**Published:** 2015-03-27

**Authors:** Sabine E Wildevuur, Lianne WL Simonse

**Affiliations:** ^1^Talma InstituteSocial SciencesVU University AmsterdamAmsterdamNetherlands; ^2^Institute for Art, Science and TechnologyCreative Care LabWaag SocietyAmsterdamNetherlands; ^3^Faculty of Industrial Design EngineeringProduct Innovation Management DepartmentDelft University of TechnologyDelftNetherlands

**Keywords:** patient-centered care, person-centered care, telemedicine, chronic disease, neoplasms, cardiovascular diseases, chronic respiratory tract diseases, diabetes mellitus, stroke, disease management, self-care, decision making, eHealth

## Abstract

**Background:**

Person-centered information and communication technology (ICT) could encourage patients to take an active part in their health care and decision-making process, and make it possible for patients to interact directly with health care providers and services about their personal health concerns. Yet, little is known about which ICT interventions dedicated to person-centered care (PCC) and connected-care interactions have been studied, especially for shared care management of chronic diseases. The aim of this research is to investigate the extent, range, and nature of these research activities and identify research gaps in the evidence base of health studies regarding the “big 5” chronic diseases: diabetes mellitus, cardiovascular disease, chronic respiratory disease, cancer, and stroke.

**Objective:**

The objective of this paper was to review the literature and to scope the field with respect to 2 questions: (1) which ICT interventions have been used to support patients and health care professionals in PCC management of the big 5 chronic diseases? and (2) what is the impact of these interventions, such as on health-related quality of life and cost efficiency?

**Methods:**

This research adopted a scoping review method. Three electronic medical databases were accessed: PubMed, EMBASE, and Cochrane Library. The research reviewed studies published between January 1989 and December 2013. In 5 stages of systematic scanning and reviewing, relevant studies were identified, selected, and charted. Then we collated, summarized, and reported the results.

**Results:**

From the initial 9380 search results, we identified 350 studies that qualified for inclusion: diabetes mellitus (n=103), cardiovascular disease (n=89), chronic respiratory disease (n=73), cancer (n=67), and stroke (n=18). Persons with one of these chronic conditions used ICT primarily for self-measurement of the body, when interacting with health care providers, with the highest rates of use seen in chronic respiratory (63%, 46/73) and cardiovascular (53%, 47/89) diseases. We found 60 relevant studies (17.1%, 60/350) on person-centered shared management ICT, primarily using telemedicine systems as personalized ICT. The highest impact measured related to the increase in empowerment (15.4%, 54/350). Health-related quality of life accounted for 8%. The highest impact connected to health professionals was an increase in clinical outcome (11.7%, 41/350). The impacts on organization outcomes were decrease in hospitalization (12.3%, 43/350) and increase of cost efficiency (10.9%, 38/350).

**Conclusions:**

This scoping review outlined ICT-enabled PCC in chronic disease management. Persons with a chronic disease could benefit from an ICT-enabled PCC approach, but ICT-PCC also yields organizational paybacks. It could lead to an increase in health care usage, as reported in some studies. Few interventions could be regarded as “fully” addressing PCC. This review will be especially helpful to those deciding on areas where further development of research or implementation of ICT-enabled PCC may be warranted.

## Introduction

Information and communication technology (ICT) offers a means to support the self-management of chronic diseases and “empowerment” of patients, primarily through the Internet. Chronic diseases—also known as noncommunicable diseases—generally progress slowly over a long time. According to the World Health Organization (WHO), the “big 5” chronic diseases are diabetes mellitus, cardiovascular and chronic respiratory diseases, cancer, and stroke [1]. In Western society, chronic diseases make up the largest proportion of diseases and this is expected to grow further as a result of an aging society putting pressure on the sustainability of the health care system. By successfully adapting to a chronic illness and self-managing the disease, people are able to handle their life with some degree of independence despite their medical condition and are capable of participating in social activities including work and feel healthy despite their limitations [2,3]. For “connected” home health care during disease management, patients are expected to increasingly use eHealth services in codecision with their health care providers and thus play an active role in managing their own disease. eHealth offers a promising way to connect chronic patients and their health care providers, thereby ensuring that both chronic patients and health care providers are more involved in the long-term care needed for chronic diseases. In-depth research has been conducted to show that patients use health-related virtual communities and electronic support groups to keep themselves informed on treatment decisions and to manage their health [4,5]. However, these ICT applications focus on situations in which a health care professional is not necessarily engaged. Moreover, the impact of these types of ICT and person-centered care (PCC) interventions on (health-related) quality of life is unknown. In our study, we explored the extent to which ICT applications have been used to support self-management of 1 of the 5 chronic diseases—in situations where both a health care professional and patient are involved—and determine their impact.

Because health systems and services have become overly biometrics-oriented, disease-focused, technology-driven, and doctor-dominated, WHO advocates putting patients at the center of health care addressing PCC as a key dimension of health care quality [6]. The 21st century is envisaged as the century of PCC, especially in the care of the chronically ill [3]. The term PCC was initially used in the field of elderly care, where practitioners sought to provide better services to particularly frail and vulnerable people. Nowadays, more and more health care professionals, policy makers, and managers envision that patients could benefit from a person-centered approach to care in which the patient is no longer the passive target of a medical intervention, but is instead actively involved in his or her care [7].

Ekman [7] distinguished 3 routines of PCC activities:

1. Initiating the partnership: patient narratives;
2. Working the partnership: shared decision making; and
3. Safeguarding the partnership: documenting the narrative.

A narrative is defined as a spoken or written account of connected events. Modern medicine is generally disease-oriented and evidence-based; PCC starts with the person’s subjective experience of his or her illness and its impact on daily life [8]. Ekman et al [7] stated that the narrative is the starting point for building a collaborative, equalitarian partnership between the provider (care and treatment expert) and the patient (person expert) that encourages and empowers patients to actively take part in finding solutions to their problems.

Initial studies on PCC are promising and suggest that an implemented PCC approach shortens hospital stays and improves quality of care [9,10]. Given the growing interest in the topic of PCC [11], the term is slowly entering the scholarly discussions around ICT interventions. eHealth—supported by ICT—could encourage patients to take an active part in their health care and the decision-making process, “empower” them, and support a person-centered approach [7]. Connecting patients and health care professionals would not only improve the (technical) system of communicating, but also trigger social innovations of care models in which new ways of interacting and deciding improve the quality and efficiency of the organization [12]. However, we do not know to what extent ICT-enabled PCC exists, has been studied, and proved to be effective in terms of medical and organizational outcomes, such as cost efficiency. In this study, we focused on identifying the gaps in this field.

With the introduction of the Internet, Web-based technology has been applied to health-related ICT systems, with an initial focus on fields such as telemedicine and telemonitoring, and more recently in Medicine 2.0 approaches applying Web 2.0 technologies [13]. Telemedicine is defined in the Medical Subject Heading (MeSH) from 1993 as follows: “Telemedicine is the delivery of health services via remote telecommunications. This includes interactive consultative and diagnostic services.” Telemonitoring represents a patient management approach combining various information technologies for monitoring patients at a distance [14]. These advances have led to reviews on the specific technology of mHealth and eHealth [15,16]. With respect to PCC, scoping reviews on care management have been conducted within the fields of reproductive medicine and chronic heart failure [9,17]. However, no such studies on the combination of ICT and PCC management have been found.

We studied ICT interventions concerning the whole range of Internet technologies introduced since the inception of the Internet in 1989, from telemedicine to the new semantic and Web-based technologies of Health 2.0 and Medicine 2.0 technologies, including the recent evolution to smartphone communication with app technologies, and how these eHealth technologies are linked to PCC and with what impact. Through shared decision making, clinicians can help patients understand the importance of the information, measurements, and preferences in making the decisions that are best for them [18]. The innovations in ICT could support PCC routines, self-management and empowerment, and enable persons to codecide about their medical treatments [19].

Given the lack of a general overview of the extent and nature of published research involving the subset of ICT interventions in PCC for chronic conditions, the aim of this study was to contribute by exploring existing studies and to draw conclusions regarding the overall state of research activities and discover research gaps. The objective of this paper is to provide a review of the literature and to scope the field with respect to 2 research questions. Firstly, which ICT interventions have been used to support patients and health care professionals in PCC management of the big 5 chronic diseases? Secondly, what is the impact of these interventions, such as on health-related quality of life and cost efficiency?

This paper addresses the methods of the scoping review and its 5 different stages. Results provides overviews of the primary studies on PCC-ICT with participation of persons with a chronic condition of diabetes mellitus, cardiovascular and chronic respiratory diseases, cancer, or stroke. The state of knowledge in the health care domain is reported in terms of volume and nature and in relation to the outcomes reported. In Discussion, the review results are interpreted and compared with prior work. In addition, theoretical and practitioner implications of the study are described.

## Methods

### Scoping Review Study

#### Overview

We employed a rigorous literature review procedure by adopting the scoping review method. This is an appropriate method to systematically scan and evaluate which studies are within or out of the scope of the research area that is explored for evidence [20,21]. We considered other types of literature review methods, such as systematic review, meta-analysis, and structured reviews, which share similar activities such as the collection, evaluation, and presentation of available research evidence in a systematic manner. However, we chose to carry out a scoping review study because it best fit our research purpose with the emphasis placed on the scoping technique to “map” relevant literature in the field of interest rather than collecting similar evidence for a highly focused research question. The method is effective in identifying gaps in the evidence base where no research has been conducted and identifying emerging results in new fields of research; the methodological framework of Arksey and O’Malley was followed [22]. Five stages of scoping and review were carried out: (1) identify the research question, (2) identify relevant studies, (3) select studies, (4) chart the data, and (5) collate, summarize, and report the results.

#### Stage 1: Identifying the Research Question

The research question was conceived from people’s high expectations regarding the potential impact of ICT innovations on heavily overburdened health care organizations, specifically in combination with an increase in self-management of diseases of long duration such as chronic diseases. We postulated that ICT could help persons with chronic conditions to interact directly with their health care providers about their personal health concerns and thereby empower them in the self-management of their personal health (information) and care plan. To search for evidence that might support our postulate, we formulated the following questions:

1. Which ICT interventions have been used to support chronic patients with the big 5 chronic conditions and their health care providers in PCC?
2. What is the impact of these interventions on health-related quality of life, and cost efficiency?
3. What other relevant study outcomes have been reported?

In the context of this paper, patients are defined as “individuals who are interacting directly with health care providers and services about personal health concerns” [23]. Starting from the point of view of ICT-enabled self-care and seeing “empowerment” as a possible outcome of applying this to the field of management of chronic diseases, we chose the definition of person-centered care coined by Ekman [7].

We consider eHealth as the use of ICT for health, as stated by the WHO initiative Global Observatory for eHealth. Our research builds on and contributes to the eHealth field, as defined by Eysenbach [22]: “eHealth is an emerging field in the intersection of medical informatics, public health, and business referring to health services and information delivered or enhanced through the Internet and related technologies. In a broader sense, the term characterizes not only a technical development, but also a state-of-mind, a way of thinking, an attitude, and a commitment for networked, global thinking to improve health care locally, regionally, and worldwide by using information and communication technology” [13].

For the purpose of our study, we initially defined PCC-ICT as a category of Internet technology that connects patients to health care professionals and enables them to interact and exchange information, including multimedia data such as audio (voice), video, and images. The PCC-ICT category covered different modes of Web communication including dedicated telemonitoring and/or telemedicine systems, Internet-based systems, telephone, and mobile phones. It excluded electronic patient record systems, public health information, and clinical and decision support systems for health care professionals only.

The outcome terms health-related quality of life and cost efficiency in the research question were only mentioned because they received considerable attention from health care managers and scholars, but in the scoping review were not restricted to these 2 outcomes. On the contrary, we conducted this scoping study to explore, extract, and describe all relevant outcomes used in the studies.

#### Stage 2: Identifying Relevant Studies

To identify original studies suitable for answering the research questions, we searched EMBASE, PubMed, and the Cochrane Library. To determine the relevant search words (keywords differ between databases), a medical information specialist devised an initial search strategy based on the research questions and definitions of key concepts, and on 10 seed articles [4,5,15,16,24-29]. The strategy was refined in the light of other published scoping review searches and other relevant sources [15,30,31]. A medical information specialist checked the final search syntaxes.

The search syntax was composed of “person-centered care,” “ICT,” and their synonyms, including the different types of spelling (US and UK), such as “person-centred care,” “self-care,” “self-management,” “e-health,” “Web 2.0,” “decision support techniques,” “videoconferencing,” “cellular phone,” “remote consultation,” “user-computer interface,” “Internet,” and “telemedicine” combined with “chronic disease,” “diabetes mellitus,” “cardiovascular,” and “chronic respiratory diseases,” “cancer,” and “stroke” and their synonyms.

Only those studies published between January 1989 and December 2013 were included. The start date of 1989 was chosen because the Internet went public in 1989. The end date was the last date on which we accessed the medical electronic databases. The search was limited to studies in English because of the costs and time involved in translating material in foreign languages such as French, German, Polish, Spanish, Russian, and Chinese. The search excluded letters, editorials, and news items. To manage the digital output from the search, we used EndNote software. The EndNote database comprised 9380 references with links to the digital portable document formats (PDFs) of the studies stored in the source database of the journals.

#### Stage 3: Study Selection

For the selection of studies, inclusion and exclusion criteria were developed and applied iteratively over 4 rounds of duplicate screening involving all authors as reviewers (Table 1).

##### First Review Step

In the first round, we screened titles and abstracts and excluded studies published before 1989, studies in which no ICT was involved, nonrelevant studies in which “mobile” was used in the sense of mobility (eg, mobile teams), nonrelevant studies focusing on mobile phones and the risk of brain damage as a result of mobile phone usage, studies on preventive care and public care involving screening and prevention activities, and studies on acute diseases (eg, acute stroke). Furthermore, studies focusing on children as the main target group were excluded because children do not manage their health on their own. Retained for inclusion were all articles addressing topics of direct relevance to the research questions: the big 5 chronic diseases, chronic care, PCC, ICT intervention, and an outcome measurement of some sort, including health-related quality of life and cost efficiency. In this round, the database was subdivided into the 5 chronic diseases.

##### Second Review Step

In the second round, the articles in each subdivision of chronic diseases in EndNote were reviewed based on title and abstract. The researchers determined whether the studies included connected-care communication of some sort that involved both the patients and their health care professionals, and ICT intervention (including telephone) to facilitate communication and interaction. In this round, we established separate folders of groups in EndNote based on our inclusion and exclusion criteria. In the separate folders, we excluded addressed applications for only health care professionals, community applications for online self-help groups, self-tests (diagnoses) for patients, and intramural health care settings. Even though we do consider that these applications are important, they do not meet our criterion that these applications should be part of an established relationship and collaboration between a patient and his or her health care professional.

##### Third Review Step

The third screen involved extracting the data by reviewing the abstracts and full text of the articles within each of the 5 databases on chronic diseases. The classification scheme for extracting the data addressed the categories: time (year of publication); origin (country); type of ICT intervention (mode of communication, data type, users); type of connected care (type of disease management, mode of PCC), and outcomes (person outcomes, health care professional outcomes, organization outcomes, and technical outcomes). General information such as gender, age, and background were not included since this scoping review study was intended to “map” relevant literature in the field of interest rather than collect evidence for a highly focused research question.

Additional criteria were developed iteratively to retain a set of articles. For example, telephones were identified as the first connection devices that made remote health care service between patient and health care provider possible. For exclusion, the criterion was developed on personal health records and other medical record applications for uses other than PCC-ICT self-management.

**Table 1:** Inclusion and exclusion criteria.

	Inclusion criteria	Exclusion criteria
Collection of studies for the research data base	Publications after the invention of Internet (1978) onwards from 1989 when the first studies have been reported on e-health, in which Internet technology is applied in the health domain	Publications before 1989
	Publications in English language	Publications in other languages than English
	Published studies in EMBASE, PubMed and Cochrane Library.	Letters, editorials, news items and conference abstracts
First review step	Persons coping with one or more of the “big five” of chronic diseases	Persons coping with an acute diseases, such as acute stroke
	Chronic Care for persons already diagnosed with a chronic disease	Preventive Care and Public Care involving screening and prevention activities.
	Person centered self management and self care involved	Children (since they are taken care of by their parents)
	ICT involved	No ICT involved in the study
		Mobile in sense of mobility (mobile teams)
		The risk on brain damage through the use of cellular phone
	Medical study relating outcomes to ICT-intervention	Managerial study outcomes of for example cost estimation comparisons, or proposed strategies, care models etc
	Theoretical study outcomes such as frameworks	
	Study outcomes measuring Health related quality of life (HRQL) and Quality of Life (QoL)	
	Study outcomes measuring Cost efficiency	
	Study outcomes measuring other impact and performance factors	
	Documenting, monitoring and interaction applications for person-centered care	
Second review step	Connected care communication: multiple target groups as users of the application	One target group of the Health care application
	Related to a person or patient	No patients mentioned or involved
	Minimal two users involved; a patient person with chronic condition and health care professional	Health care professional applications
		Patient community applications
	Home health care setting: care activities at home connected to care activities at other health care settings	Care only in a hospital or other intramural setting
		Self tests at home (for example, self-diagnosis)
Third review step	Telephone as device to connect patients with caregivers, in combination with remote health care services	
	Share personal health concerns	
	Manage own personal health information	Personal health records and other medical records applications for other usage
	Manage personal care plan	
	Virtual Reality for rehabilitation of stroke	
	Twitter	
	Skype	

##### Fourth Review Step

This step involved analyzing the systematic reviews to determine which studies had been carried out in an evidence-based manner, meeting the inclusion and exclusion criteria of the scoping review. Some of the studies that were described as “a systematic review” did not in fact meet the criteria set for a systematic review. However, these studies were not excluded because a scoping review does not assess the quality of the studies [22].

An additional exclusion criterion was that publications that did not include a full study (ie, ones that consisted merely of protocols or structured abstracts) were left out. Another additional inclusion criterion concerned technologies that were incidentally described within the searched databases. These included the usage of Skype, social media such as Twitter, or robot assistance for rehabilitation. We expect these applications—and others such as wearable devices—to be studied and described more frequently in the years to come and thus included them in the existing list of criteria.

The reviewers met a couple of times at every reviewing round to discuss the selection of studies and to refine the inclusion and exclusion criteria. The criteria were used in an iterative way, meaning that where necessary the reviewing procedure was repeated to ensure that the references were covered in a comprehensive way.

#### Stage 4: Charting the Data

For the critical fourth stage, we carefully crafted the classification schemes in such a way that ICT interventions used for PCC were classified into mutually exclusive and cumulatively exhaustive categories. This required a number of iterations in refinement and modification of the categories to ensure reliability of the study classification.

To answer the research questions, we created charts for:

1. Study context identification: time and geographical origin of the study;
2. Process intervention studied: types and modes of ICT intervention used for connected-care activities;
3. Per targeted population of patients with a chronic condition: the monitoring, documenting, and interacting devices per connected-care activity; and
4. Study outcome measures.

We coded ICT interventions into 4 categories: (1) telephone-based, (2) mobile phone–based, (3) Internet-based, and (4) dedicated telemonitoring/telemedicine system–based. Distinctions in applications for each of these types of hardware and software were made according to their data source (telephone, smartphone, Internet, or telemonitoring/telemedicine) and their primary function: documenting, interacting, and/or monitoring. In this stage, we compared the studies first within the chronic care management domain and then across the different chronic disease domains. We undertook this process manually using tabulation charts in Excel as visual aids. Each study was charted in a table per chronic care management activity and the ICT intervention for either activity to connect disease management and/or support person–centered activities. We determined patterns of commonalities and differences among the ICT interventions and care management activities.

#### Stage 5: Collating, Summarizing, and Reporting the Results

Having charted the information, we were able to numerically analyze the included studies. We then answered the research questions based on the analysis overviews. Through the systematic reporting and charting of the data, we were also able to make comparisons across ICT interventions, identify contradictory evidence regarding specific interventions, and identify research gaps in the existing research evidence.

## Results

### Overview

**Figure 1:**
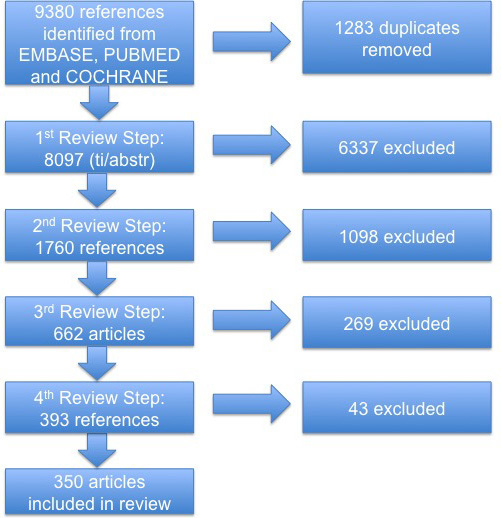
Search and screening results.

From the initial 9380 search results from EMBASE (n=6702), PubMed (n=1866), and the Cochrane Library (n=812), we identified 350 studies that qualified for inclusion (Figure 1). Classified according to the participation of persons with a chronic condition, the number of studies by condition was as follows: diabetes mellitus (n=103), cardiovascular disease (n=89), chronic respiratory disease (n=73), cancer (n=67), and stroke (n=18) (acute stroke was excluded) (Multimedia Appendix 1).

First, we excluded the duplicates (n=1283). In the first screening round, we excluded both the ones published before 1989 and nonrelevant studies (n=6337) according to the exclusion criteria we developed in this round (see Figure 1), leaving 1760 studies.

In the second screening round, 1098 articles appeared not to address the research scope in terms of the inclusion criteria. In the third screening round, 269 were excluded, leading to 393 articles. Finally, in the fourth screening round, we charted the systematic reviews from the separate EndNote folder from the 105 systematic reviews; 62 were eventually included and 43 excluded. We were left with 350 studies.

### Study Characteristics

In characterizing the included studies by origin, 40 countries are represented: Europe (147/350, 42.0%), North America (138/350, 39.4%), Pacific region (39/350, 11.1%), Asia (20/350, 5.7%), Middle East (8/350, 2.3%), and Latin America (3/350, 0.9%) (see Table 2).

**Figure 2:**
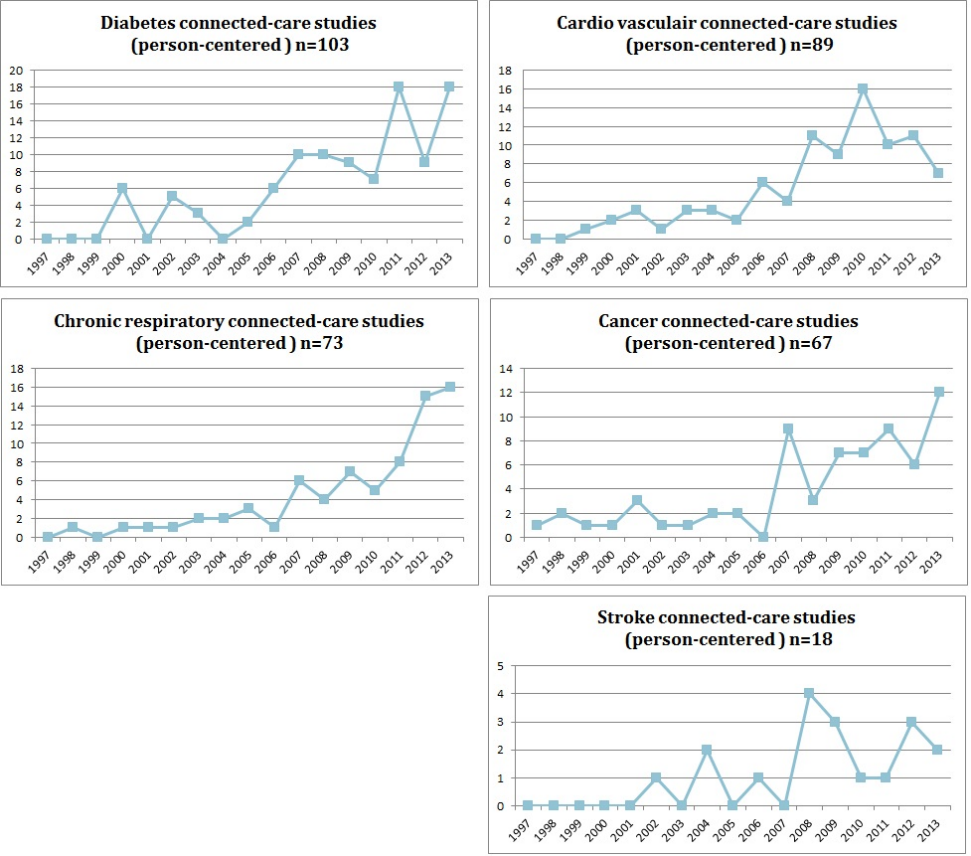
Number of studies conducted over the years for the “big five” chronic conditions.

Figure 2 shows that almost 10 years after the starting point of our search strategy (1989), the number of studies published annually increased until 2013. Within the domain of cancer, the first studies were identified on connected care in 1997. Around 2005, attention to connected care seemed to rise, with an even more substantial increase in publication volume from 2010 onward. The trend lines differed for the big 5 chronic conditions, with chronic respiratory conditions showing the steadiest increase and the others more fluctuation; cardiovascular conditions showed a slight decrease in recent years.

**Table 2:** Representation of the studies according to geographical location.^a^

Continent and country		Overall, n (%)	Chronic disease, n (%)				
			Diabetes	Cardiovascular	Chronic respiratory	Cancer	Stroke
		N=350	n=103	n=89	n=73	n=67	n=18
**Europe**							
	Netherlands	24 (6.9)	5 (1.4)	5 (1)	10 (3)	4 (1)	
	Belgium	1 (0.3)		1 (0)			
	United Kingdom	47 (13.4)	10 (2.9)	11 (3)	14 (4)	12 (3)	
	Germany	17 (4.9)	5 (1.4)	10 (3)	1 (0)		1 (0)
	France	3 (0.9)	1 (0.3)	1 (0)		1 (0)	
	Italy	21 (6.0)	5 (1.4)	7 (2)	4 (1)	1 (0)	4 (1)
	Poland	6 (1.7)	3 (0.9)	3 (1)			
	Austria	1 (0.3)	1 (0.3)				
	Switzerland	2 (0.6)		1 (0)			1 (0)
	Spain	8 (2.3)	1 (0.3)	3 (1)	3 (1)	1 (0)	
	Denmark	5 (1.4)			5 (1)		
	Finland	3 (0.9)	1 (0.3)			1 (0)	1 (0)
	Sweden	3 (0.9)				3 (1)	
	Portugal	1 (0.3)			1 (0)		
	Cyprus	1 (0.3)		1 (0)			
	Norway	4 (1.1)		2 (1)		2 (1)	
	Total	147 (42.0)	32 (9.1)	45 (12)	38 (11)	25 (7)	7 (2)
**North America**							
	United States	116 (33.1)	48 (13.7)	23 (7)	13 (4)	29 (8)	3 (1)
	Canada	22 (6.3)	2 (0.6)	9 (3)	7 (2)	3 (1)	1 (0)
	Total	138 (39.4)	50 (14.3)	32 (9)	20 (6)	32 (9)	4 (1)
**Asia**							
	China	4 (1.1)	2 (0.6)		2 (1)		
	Taiwan	5 (1.4)	2 (0.6)		2 (1)		1 (0)
	Korea	4 (1.1)	3 (0.9)			1 (0)	
	Thailand	1 (0.3)	1 (0.3)				
	Hong Kong	3 (0.9)			1 (0)		2 (1)
	Japan	1 (0.3)		1 (0)			
	India	1 (0.3)		1 (0)			
	Sri Lanka	1 (0.3)	1 (0.3)				
	Total	20 (5.7)	9 (2.6)	2 (1)	5 (1)	1 (0)	3 (1)
**Middle East**							
	Saudi Arabia	2 (0.6)	1 (0.3)	1 (0)			
	Israel	4 (1.1)	1 (0.3)	3 (1)			
	Iran	2 (0.6)	2 (0.6)				
	Total	8 (2.3)	4 (1.1)	4 (1)			
**Latin America**							
	Chile	1 (0.3)	1 (0.3)				
	Brazil	1 (0.3)	1 (0.3)				
	Panama	1 (0.3)	1 (0.3)				
	Total	3 (0.9)	3 (0.9)				
**Pacific**							
	Australia	37 (10.6)	5 (1.4)	9 (3)	10 (3)	9 (3)	4 (1)
	New Zealand	2 (0.6)		1 (0)	1 (0)		
	Total	39 (11.1)	5 (1.4)	10 (3)	11 (3)	9 (3)	4 (1)

^a^ More than 1 country possible due to consortia (n=5). Percentages estimated by total number of studies (N=350).

### Information and Communication Technology Enabling Person-Centered Care for Diabetes Mellitus

From the total number of relevant studies concerning PCC in which patients with a diabetes condition were central (n=66), ICT applied for self-body measurement (eg, with a glucose meter device and monitoring system) was the most used (48/66, 47%) (Table 3).

The second in line was personal lifestyle sharing (16/103, 15.5%), which is distinctively related to diabetes care. ICT for shared treatment decisions ranks low, with 2 studies found (2/103, 1.9%). In broader terms, connected care for diabetes, the most studied ICT intervention, addressed education with transfer of diabetes knowledge from the care provider to the patient (26/103, 25.2%).

The most commonly used ICT intervention employed telemonitoring/telemedicine systems (42/103, 40.8%). Internet interventions ranked second (29/103, 28.2%), whereas mobile phone interventions accounted for 24.3% (25/103) and telephone interventions for 16.5% (17/103). Interestingly, text messaging was used in 9.7% (10/103) of the studies. Glucose monitoring devices plus systems ranked third (13/103, 12.6%). These and other modes of telemedicine systems were mostly used (21/103, 20.2%) for the PCC activity of self-body measurement versus 9% used for physical care with measurement by the physician. Within the category of mobile phone interventions, 9% used a monitoring app. A personal e-diary app is worth mentioning as a personal means of sharing health information with the care provider. Within the category of Internet interventions, most of the interventions concerned monitoring, but were integrated with interaction via the Web app (6%). Another type of Web-based app used more than once was a documenting Web app that was mostly employed for sharing lifestyle information with the health care professional. The last category of charted ICT intervention studies indicated a high research interest (17%) in low-tech technology interventions via telephone, the first connecting device enabling remote care management by means of follow-up telephone calls made by nurses.

### Information and Communication Technology Enabling Person-Centered Care for Chronic Cardiovascular Diseases

The studies in which cardiovascular patients participated showed a clear preference (71%, 63/89) for telemonitoring/telemedicine system interventions applied for PCC, self-measurement of the body (38%, 34/89), versus interventions applied for connected physical care (17%), and education (9%) as a way to connect the patient and the health care professionals providing advice and service (see Table 4). In almost one-quarter of the studies (23%, 21/89), telephone interventions—in particular nurse telephone calls (21%, 19/89)—were used.

Of the 89 studies in the scope of cardiovascular conditions, the most studied person-centered care activity was self-measurement of the body (38%, 34/89). We discovered 3 studies addressing self-rehabilitation exercises by a virtual clinic app on the Internet and a telemonitoring system. A telemedicine system was used for shared treatment decision making in only 1% (1/89) of the studies.

Another 8% (7/89) of the studies dealt with the use of remote monitoring and a cardiac implant device for self-measurement of the body. These are remote monitoring apps for implanted cardiac pacing systems, which enable persons and health care professionals to (self) monitor the heart implant and are used specifically for cardiovascular patients. Mobile phone interventions were used in 8% (7/89) of the studies; in 7% (6/89) of the studies, monitoring apps were used to self-measure the body, whereas 1% (1/89) were used for educational purposes. We also found combinations of interventions (eg, telephone support by nurses for the educational part and a monitoring device with a Web app for self-monitoring and sharing the data with health professionals).

**Table 3:** Information and communication technology intervention used for person-centered care for diabetes (n=103).^a^

ICT Intervention		Overall, n (%)	Connected-care activity, n (%)				Person-centered care activity, n (%)		
			Consult	Physical care	Behavior therapy	Education	Self-measurement	Lifestyle sharing	Shared decisions
		n=103	n=3	n=8	n=10	n=26	n=48	n=16	n=2
**Telephone intervention**		17 (16.5)		1 (1)	2 (2)	4 (4)	6 (6)	4 (4)	
	Nurse telephone calls	8 (7.8)		1 (1)	2 (2)		2 (2)	3 (3)	
	Automated telephone calls	8 (7.8)				4 (4)	4 (4)		
	Nurse call center	1 (1.0)						1 (1)	
**Mobile phone intervention**		25 (24.3)	1 (1)		2 (2)	6 (6)	11 (11)	5 (5)	
	Smartphone calls	1 (1.0)					1 (1)		
	Text messaging	10 (9.7)			1 (1)	4 (4)	2 (2)	3 (3)	
	Video messages	1 (1.0)				1 (1)			
	e-Dairy messaging app	2 (1.9)					1 (1)	1 (1)	
	Monitoring app	9 (8.7)				1 (1)	7 (7)	1 (1)	
	Interaction app	2 (1.9)	1 (1)		1 (1)				
**Internet intervention**		29 (28.2)	2 (2)	2 (2)	3 (3)	7 (7)	10 (10)	4 (4)	1 (1)
	Secure messaging app	1 (1.0)	1 (1)						
	Health knowledge base	3 (2.9)				2 (2)			1 (1)
	Documenting app	4 (3.9)			1 (1)			3 (3)	
	Personal health record app	3 (2.9)			1 (1)	1 (1)	1 (1)		1 (1)	1 (1)		
	Monitoring app	3 (2.9)					3 (3)		
	Monitoring device + app	2 (1.9)		1 (1)		1 (1)			
	Interaction app	3 (2.9)	1 (1)				1 (1)	1 (1)	
	Monitoring + interaction app	6 (5.8)		1 (1)		2 (2)	3 (3)		
	Monitoring video conferencing	2 (1.9)					2 (2)		
	Virtual clinic	2 (1.9)			1 (1)	1 (1)			
**Telemedicine intervention**		42 (40.8)		5 (5)	3 (3)	9 (9)	21 (20)	3 (3)	1 (1)
	Video phone visits	1 (1.0)				1 (1)			
	Monitoring device + system	13 (12.6)		1 (1)			10 (10)	2 (2)	
	Telemonitoring system	7 (6.8)		2 (2)			5 (5)		
	Telemedicine system	21 (20.4)		2 (2)	3 (3)	8 (8)	6 (6)	1 (1)	1 (1)

^a^ More than 1 (person-centered) connected-care management activity possible. Percentage estimated by total number of studies (n=103).

**Table 4:** Information and communication technology intervention used for person-centered care for chronic cardiovascular conditions (n=89).^a^

ICT Intervention		Overall, n (%)	Connected-care activity, n (%)				Person-centered care activity, n (%)				
			Consult	Medication	Physical care	Education	Self-measurement	Rehabilitation exercises	Lifestyle sharing	Shared decisions	Self-care plan
		n=89	n=10	n=3	n=19	n=20	n=47	n=3	n=1	n=1	n=1
**Telephone intervention**		21 (24)	5 (6)	1 (1)	4 (4)	8 (9)	2 (2)		1 (1)		
	Nurse telephone calls	19 (21)	4 (4)	1 (1)	3 (3)	8 (9)	2 (2)		1 (1)		
	Nurse call center	2 (2)	1 (1)		1 (1)						
**Mobile phone intervention**											
	Monitoring app	7 (8)				1 (1)	6 (7)				
**Internet intervention**		13 (15)	3 (3)	1 (1)		3 (3)	5 (6)	1 (1)			
	Secure messaging app	1 (1)				1 (1)					
	Health knowledge base	2 (2)				2 (2)					
	Documenting app	1 (1)	1 (1)								
	Personal health record app	1 (1)	1 (1)								
	Monitoring app	3 (3)		1 (1)			2 (2)				
	Monitoring device + app	1 (1)					1 (1)				
	Monitoring + interaction application	3 (3)	1 (1)				2 (2)				
	Virtual clinic	1 (1)						1 (1)			
**Telemedicine intervention**		63 (71)	2 (2)	1 (1)	15 (17)	8 (9)	34 (38)	2 (2)		1 (1)	1 (1)
	Monitoring system + cardiac implant	7 (8)					7 (8)				
	TV channel system	1 (1)				1 (1)					
	Telemonitoring system	33 (37)	1 (1)		13 (15)	1 (1)	16 (18)	2 (2)			
	Telemedicine system	22 (25)	1 (1)	1 (1)	2 (2)	6 (7)	11 (12)			1 (1)	1 (1)

^a^ More than 1 (person-centered) connected-care management activity possible. Percentage estimated by total number of studies (n=89).

### Information and Communication Technology Enabling Person-Centered Care for Chronic Respiratory Conditions

Within the group of studies addressing patients with chronic respiratory disease (n=73) (Table 5), more than half of the studies (52%, 38/73) used Internet interventions; this was the highest score among the 5 categories of chronic diseases and much higher than in the category of studies addressing cardiovascular patients (13%), for example. In 28% (21/73) of the studies, telemonitoring/telemedicine system interventions were used. One-fifth (20%, 15/73) used telephone interventions with nurse telephone calls accounting for 16% (12/73) and mobile phones for 14% (11/73). Furthermore, we extracted and added Skype as a new Web intervention (1%, 1/73).

Concerning person-centered care, we found 2 studies (3%, 2/73) in which a self-care plan was created and decided on, 1 with a monitoring videoconference app on the Internet and 1 with a telemedicine system. Also noteworthy, only 5% (4/73) of the studied telemedicine system interventions were used for consultations and few (7%, 5/73) of the telephone interventions focused on physical care or education. This indicates that the transformation to PCC is still in its early phase in disease management for chronic respiratory diseases. Similar to the category of patients with cardiovascular diseases, the category of patients with chronic respiratory diseases also had an additional PCC activity—compared to the other groups of chronic patients—namely exercises for self-rehabilitation, which is of importance for this group; 8% (6/73) of the studies were targeted toward this type of activity.

**Table 5:** Information and communication technology interventions used for person-centered care for chronic respiratory conditions (n=73).^a^

ICT Intervention		Overall, n (%)	Connected-care activity, n (%)					Person-centered care activity, n (%)		
			Consult	Medication	Physical care	Behavior therapy	Education	Self-measurement	Rehabilitation exercises	Self-care plan
		n=73	n=5	n=3	n=7	n=4	n=12	n=46	n=6	n=2
**Telephone intervention**		15 (21)			6 (8)	2 (3)	5 (7)		2 (3)	
	Nurse telephone calls	12 (16)			4 (5)	2 (3)	5 (7)		1 (1)	
	Automated telephone calls	2 (3)			1 (1)				1 (1)	
	Nurse call center	1 (1)			1 (1)					
**Mobile phone intervention**		11 (15)		1 (1)		1 (1)	2 (3)	6 (8)	1 (1)	
	Text messaging	3 (4)		1 (1)			1 (1)	1 (1)		
	Monitoring app	6 (8)				1 (1)	1 (1)	4 (5)		
	Music app	1 (1)							1 (1)	
	Interaction app	1 (1)						1 (1)		
**Internet intervention**		38 (52)	1 (1)	2 (3)	1 (1)	1 (1)	5 (7)	26 (36)	1 (1)	1 (1)
	Health knowledge base	3 (4)				1 (1)	2 (3)			
	Documenting app	1 (1)		1 (1)						
	Personal health record app	1 (1)			1 (1)					
	Monitoring app	4 (5)						4 (5)		
	Monitoring device + app	14 (19)		1 (1)				12 (16)	1 (1)	
	Monitoring + interaction app	13 (18)	1 (1)				3 (4)	9 (12)		
	Monitoring video conferencing	1 (1)								1 (1)
	Skype video app	1 (1)						1 (1)		
**Telemedicine intervention**		21 (29)	4 (5)					14 (19)	2 (3)	1 (1)
	Monitoring device + system	1 (1)	1 (1)							
	Telemonitoring system	8 (11)	1 (1)					6 (8)	1 (1)	
	Telemedicine system	12 (16)	2 (3)					8 (11)	1 (1)	1 (1)

^a^ More than 1 (person-centered) connected-care management activity possible. Percentage estimated by total number of studies (n=73).

### Information and Communication Technology Enabling Person-Centered Care for Cancer

The category of cancer care was the broadest one, with ICT interventions used and studied for a wide variety of self-care and connected-care activities (Table 6). Cancer care management appeared to be one of the most progressive ones in our findings, as involved cancer patients actively used the latest ICT innovations and the highest number of studies (n=23) related to shared management activities in PCC (excluding self-measurement of body activities that are not typically used among those with cancer). Only here did we find studies in which patients and their care providers used the social medium Twitter for PCC for social support.

**Table 6:** Information and communication technology interventions used for person-centered care for cancer (n=67).^a^

ICT Intervention		Overall, n (%)	Connected-care activity, n (%)						Person-centered care activity, n (%)						
			Consult	Medication	Physical care	Behavior therapy	Palliative care	Education	Self-measurement	Knowledge sharing	Self-reporting symptoms	Lifestyle sharing	Shared decisions	Self-care plan	Social support
	n=67	n=12	n=4	n=12	n=4	n=1	n=17	n=1	n=5	n=5	n=4	n=3	n=3	n=2
**Telephone intervention**		16 (24)	4 (6)	1 (1)	1 (1)	2 (3)		4 (6)				3 (4)		1 (1)	
	Nurse telephone calls	15 (22)	4 (6)		1 (1)	2 (3)		4 (6)				3 (4)		1 (1)	
	Automated telephone calls	1 (1)		1 (1)											
**Mobile phone intervention**		8 (12)		1 (1)	4 (6)						1 (1)		1 (1)		1 (1)
	Monitoring app	6 (9)		1 (1)	3 (4)						1 (1)		1 (1)		
	Interaction app	1 (1)			1 (1)										
	Twitter	1 (1)													1 (1)
**Internet intervention**		30 (45)		2 (3)	2 (3)	2 (3)	1 (1)	9 (13)	1 (1)	4 (6)	4 (6)	1 (1)	1 (1)	2 (3)	1 (1)
	Health knowledge base	9 (13)			1 (1)	1 (1)		4 (6)		2 (3)					1 (1)
	Documenting app	1 (1)									1 (1)				
	Personal health record app	6 (9)		1 (1)				2 (3)			1 (1)	1 (1)		1 (1)	
	Monitoring device + app	1 (1)					1 (1)								
	Interaction app	2 (3)				1 (1)		1 (1)							
	Monitoring + interaction app	7 (10)		1 (1)	1 (1)				1 (1)		2 (3)		1 (1)	1 (1)	
	Skype video app	1 (1)						1 (1)							
	Virtual clinic	1 (1)						1 (1)							
	Internet support groups	2 (3)								2 (3)					
**Telemedicine intervention**		20 (30)	8 (12)		5 (7)			4 (6)		1 (1)			1 (1)		
	Video phone visits	3 (4)	3 (4)												
	Monitoring device + system	1 (1)											1 (1)		
	Telemedicine system	16 (24)	5 (7)		5 (7)			4 (6)		1 (1)					1 (1)

^a^ More than 1 (person-centered) connected-care management activity possible. Percentage estimated by total number of studies (n=67).

The most studied interventions for persons with a cancer condition (n=67) addressed Internet interventions for educational purposes (12%, 8/67) of connected care, especially Web portals (6%, 4/67) versus 7% (5/67) of sharing cancer knowledge in a person-centered way. This differs from the other conditions, where intervention for self-measurement of the body ranked the highest. An explanation could be that body measurements related to cancer are performed by health professionals (for the most part), illustrated by our findings that 7% (5/67) of the studied telemedicine interventions focused on physical care. A new type of PCC activity emerged from our data: 5% of the Internet interventions were applied for self-reporting symptoms. This self-care activity was distinctive and not found among studies concerning the other types of chronic diseases. A monitoring app on mobile phones, a documenting app, a personal health record app, and 2 monitoring plus interaction apps supported the self-reporting of symptoms. Overall, the total number of PCC activities (n=23) included 3 studies on shared decision making and 3 studies on self-care plan creation. Compared to connected-care activities, nurse telephone calls were used in several cases, both for consultation (6%, 4/67) and education (6%, 4/67) versus PCC personal lifestyle sharing (4%, 3/67) and cancer knowledge sharing (6%, 4/67).

### Information and Communication Technology Enabling Person-Centered Care for Stroke

The fewest studies of all the chronic condition conditions were encountered in the stroke category (n=18) (Table 7). Six studies addressed PCC, of which 5 studies supported a new distinctive type of care activity: self-rehabilitation therapy. Interestingly, high-tech innovations involving a robot assistant and virtual reality gaming were studied for self-rehabilitation. Even though stroke is regarded, according to the WHO, as one of the 5 leading chronic diseases, many of the studies on connected care by applying ICT are conducted in the field of acute stroke and mobile teams. We only included the studies when acute stroke turned into a chronic situation. Most studied ICT interventions were telemedicine/telemonitoring systems (12/18, 67%) addressing physical care, consultation, and education. A note on the reliability of this data: not all 12 studies were precise in what type of ICT intervention was used and some referred to it in very general terms, such as “telemedicine system.” We suggest keeping this in mind when interpreting these results.

**Table 7:** Information and communication technology interventions used for person-centered care for stroke (n=18).^a^

ICT Intervention		Overall, n (%)	Connected-care activity, n (%)					Person-centered care activity, n (%)	
			Consult	Physical care	Behavior therapy	Cognitive therapy	Education	Self- measurement body	Rehabilitation exercises
		n=18	n=2	n=6	n=3	n=1	n=3	n=1	n=5
**Telephone intervention**									
	Nurse telephone calls	1 (6)			1 (6)				
**Mobile phone intervention**									
	Video messages	1 (6)			1 (6)				
**Internet intervention**		7 (39)							3 (17)
	Monitoring device + app	1 (6)						1 (6)	
	Monitoring video conferencing	4 (22)		1 (6)	1 (6)		1 (6)		1 (6)
	Gaming (virtual reality)	2 (11)							2 (11)
**Telemedicine intervention**		12 (67)	2 (11)	5 (28)		1 (6)	2 (11)		2 (11)
	Robot assistant	1							1 (6)
	Telemonitoring system	4 (22)		4 (22)					
	Telemedicine system	7 (39)	2 (11)	1 (6)		1 (6)	2 (11)		1 (6)

^a^ More than 1 (person-centered) connected-care management activity possible. Percentage estimated by total number of studies (n=18).

### Outcomes

#### Overview

In addition to the (health-related) quality of life and costs efficiency outcomes, we found 33 outcome indicators (see Table 8). Extracted from the studied text, we classified these outcome indicators under 4 category definitions. We defined person outcomes (12 indicators), connected to health professional outcomes (7 indicators), organization outcomes (9 indicators), technical outcomes (5 indicators), and no outcomes. This last category included 7% (25/350) of the studies, in which no outcome measurement was found.

**Table 8:** Outcomes of the information and communication technology interventions for person-centered care and connected-care management.

Outcomes		Overall N=350		Diabetes n=103		Cardiovascular n=89		Respiratory n=73		Cancer n=67		Stroke n=18	
		Pos	Neg	Pos	Neg	Pos	Neg	Pos	Neg	Pos	Neg	Pos	Neg
		n (%)	n (%)	n (%)	n (%)	n (%)	n (%)	n (%)	n (%)	n (%)	n (%)	n (%)	n (%)
No outcomes		25 (7)		11 (3)		6 (2)		3 (1)		5 (1)			
**Person outcomes**		224 (64)	45 (13)	79 (23)	9 (3)	45 (13)	12 (3)	45 (13)	19 (5)	42 (12)	4 (1)	13 (4)	1 (0)
	Quality of life	46 (13)	6 (2)			28 (8)	1 (0)	8 (2)	3 (1)	8 (2)	2 (1)	2 (1)	
	Health-related quality of life	28 (8)	18 (5)	8 (2)	5 (1)	3 (1)		14 (4)	12 (3)	3 (1)	1 (0)		
	Mental health–related quality of life	1 (0)		1 (0)									
	Mortality (less)	1 (0)	11 (3)			1 (0)	11 (3)						
	Self-efficacy	19 (5)	4 (1)	8 (2)	1 (0)	5 (1)		1 (0)	2 (1)	3 (1)	1 (0)	2 (1)	
	Empowerment (self-care)	54 (15)	2 (1)	22 (6)		7 (2)		11 (3)	2 (1)	11 (3)		3 (1)	
	Physical condition	49 (14)		30 (9)				10 (3)		5 (1)		4 (1)	
	Metabolic control	9 (3)	1 (0)	9 (3)	1 (0)								
	Pain reduction	1 (0)								1 (0)			
	Behavior change	3 (1)		1 (0)		1 (0)		1 (0)					
	Mental health condition	13 (4)	2 (1)		2 (1)					11 (3)		2 (1)	
	Loneliness		1 (0)										1 (0)
**Connected to health professional outcomes**		81 (23)	12 (3)	11 (3)	3 (1)	32 (9)	1 (0)	23 (7)	7 (2)	6 (2)	0 (0)	9 (3)	1 (0)
	Medication adherence	12 (3)		3 (1)		5 (1)		4 (1)					
	Treatment adherence	8 (2)				4 (1)		3 (1)				1 (0)	
	Clinical outcomes	41 (12)	8 (2)	5 (1)	3 (1)	17 (5)		11 (3)	4 (1)	1 (0)		7 (2)	1 (0)
	Effectives of intervention		4 (1)				1 (0)		3 (1)				
	Documentation quality	5 (1)				1 (0)		1 (0)		2 (1)		1 (0)	
	Communication quality	9 (3)		3 (1)		2 (1)		2 (1)		2 (1)			
	Health knowledge	6 (2)				3 (1)		2 (1)		1 (0)			
**Organization outcomes (care model)**		73 (21)	59 (17)	18 (5)	6 (2)	26 (7)	31 (9)	12 (3)	19 (5)	11 (3)	3 (1)	6 (2)	0 (0)
	Cost efficiency	38 (11)	6 (2)	9 (3)	1 (0)	15 (4)	1 (0)	7 (2)	4 (1)	7 (2)			
	(Time) efficiency	11 (3)		5 (1)		4 (1)				1 (0)		1 (0)	
	Quality effectiveness	4 (1)	1 (0)	1 (0)		2 (1)		1 (0)	1 (0)				
	Productivity	1 (0)								1 (0)			
	Less hospitalization	6 (2)	43 (12)		2 (1)	2 (1)	26 (7)	4 (1)	13 (4)		2 (1)		
	Reduced comanagement	1 (0)				1 (0)							
	Implementation enablers / barriers (including ethical)	6 (2)	6 (2)	2 (1)	3 (1)	2 (1)	2 (1)		1 (0)			2 (1)	
	Improve office visits / replace face-to-face consult	1 (0)	3 (1)	1 (0)			2 (1)				1 (0)		
	Improve access difficulties	5 (1)								2 (1)		3 (1)	
**Technical outcomes**		91 (26)	2 (1)	19 (5)	0 (0)	22 (6)	0 (0)	22 (6)	1 (0)	23 (7)	1 (0)	5 (1)	0 (0)
	Feasibility	35 (10)		7 (2)		6 (2)		9 (3)		10 (3)		3 (1)	
	Usability	21 (6)		5 (1)		6 (2)		4 (1)		6 (2)			
	Satisfaction	28 (8)	1 (0)	7 (2)		7 (2)		8 (2)		5 (1)	1 (0)	1 (0)	
	Safety	6 (2)				2 (1)		1 (0)		2 (1)		1 (0)	
	Commercial feasibility	1 (0)	1 (0)			1 (0)			1 (0)				
Total outcomes: positive impact		469 (134)	118 (34)	127 (36)	18 (5)	125 (36)	44 (13)	102 (29)	46 (13)	82 (23)	8 (2)	33 (9)	2 (1)

#### Person Outcomes

A total of 15.4% (54/350) of the studies measured a positive impact on empowerment (self-care) closely followed by improvement in physical condition (14.0%, 49/350). The increase in quality of life and health-related quality of life accounted for 13.1% (46/350) and 8.0% (28/350), respectively, and self-efficacy for 5.1% (18/350).

Three person outcome indicators were found to be distinctive for one of the 5 chronic conditions: metabolic control was measured in 10 diabetes studies and lower mortality in 11 cardiovascular studies, whereas improvement in mental health was reported in 11 cancer studies and 2 stroke studies. Overall, 76.9% (269/350) of the studies reported on person outcomes, with 64.0% (224/350) reporting a positive impact versus 12.9% (45/350) reporting a negative or no impact. These findings confirm the importance of measuring the person-centeredness of the ICT intervention, for which these 5 outcome indicators are currently commonly used.

#### Connected to Health Professional Outcomes

The impact for being connected to the health care professional by ICT was found to be the highest on a familiar clinical outcomes indicator. Of the total studies, 11.7% (41/350) reported an increase in clinical outcome versus a decrease in 2.0% (7/350) of the studies. Interestingly, “medication adherence” and “treatment adherence” emerged as outcome indicators in a few studies. In relation to PCC, a few other studies suggested that “documentation quality” and “communication quality” should be used to measure the concept of acquiring better insight into the patient. Overall, one-quarter of the studies (93/350, 26.6%) reported on professional outcomes connected to health, with 23.1% (81/350) reporting a positive impact versus 3.4% (12/350) reporting a negative or no impact.

#### Organization Outcomes

Remarkably, the most studied impact on organization outcome was not cost efficiency itself, but the related impact of less hospitalization (43/350, 12.3%), closely followed by cost efficiency (38/350, 10.9%). Time efficiency was a third outcome indicator appearing in a few studies (11/350, 3.1%). Overall, 37.7% (132/350) of the studies on connected-care and person-centered ICT interventions reported on organization outcomes. To a certain extent a positive impact was reported (20.9%, 73/350), which was challenged by a relatively large number of studies that reported a negative outcome; 59 studies (16.9%) reported a negative impact regarding organization outcomes. Most reported were both a decrease in cost efficiency (1.7%, 6/350) and an increase in hospitalization (1.7%, 6/350).

#### Technical Outcomes

As far as technical outcomes related to the implementation of the ICT innovation were concerned, the most measured outcome was technical feasibility (10.0%, 35/350) followed by satisfaction (8.0%, 28/350) with the ICT intervention. Important for PCC, usability was measured in 6.0% of the studies (21/350).

In sum, a positive outcome indicator was reported 469 times (134%) versus a negative outcome indicator 118 times (34%). As a percentage of the total 350 studies, we found a relatively more positive impact in studies on diabetes (36.3%, 127/350) and cancer conditions (23.4%, 82/350) versus a relatively more negative impact in the studies on cardiovascular (12.6%, 44/350) and chronic respiratory conditions (13.1%, 46/350).

## Discussion

### Principal Findings

Shared decision making, personal information sharing, and setting up a care plan enabled by ICT seem to be relatively new. This indicates that the state of knowledge in the PCC field of interest is still emerging, meaning there are many research opportunities to contribute. The type of ICT mostly used by persons with a chronic condition for interacting with health care providers is ICT for self-measurement of the body (n=143) (Table 9); the highest rankings were found in studies on diabetes (n=48) and cardiovascular (n=47) and chronic respiratory diseases (n=46). These are in striking contrast with the lowest ranking; only 1 study was found on self-measurement of the body within the group on cancer and only 1 on stroke. Given these types of chronic diseases (cancer and stroke), physical measurements and check-ups likely require the health care provider to use specialized professional equipment. Instead, shared care management activities are enabled by the person-centered ICT for cancer (n=23) and stroke (n=5). Overall, we found 60 studies (17%) on this type of shared decision-making ICT. Cancer ranked first in number of studies followed by diabetes (n=18).

We note that hardly any of these interventions could be regarded as “fully” addressing the 3 routines of PCC for activities related to initiating the partnership (patient narratives), working the partnership (shared decision making), and safeguarding the partnership (documenting the narrative) [7].

**Table 9:** Person-centered care and information and communication technology interventions used for the big 5 chronic connected-care activities (CCA), person-centered self-measurement (PCM), and person-centered shared management (PCS).^a^

PCC-ICT interventions used		Total	Telephone	Mobile phone app	Internet app	Telemedicine system
**Overall**		398 (114)	70 (20)	52 (15)	117 (33)	159 (45)
	CCA	195 (56)	51 (15)	20 (6)	50 (14)	74 (21)
	PCM	143 (41)	8 (2)	23 (7)	43 (12)	69 (20)
	PCS	60 (17)	11 (3)	9 (3)	24 (7)	16 (5)
**Diabetes**						
	CCA	47 (13)	7 (2)	9 (3)	14 (4)	17 (5)
	PCM	48 (14)	6 (2)	11 (3)	10 (3)	21 (6)
	PCS	18 (5)	4 (1)	5 (1)	5 (1)	4 (1)
**Cardiovascular**						
	CCA	52 (15)	18 (5)	1 (0)	7 (2)	26 (7)
	PCM	47 (13)	2 (1)	6 (2)	5 (1)	34 (10)
	PCS	6 (2)	1 (0)	0 (0)	1 (0)	4 (1)
**Chronic respiratory**						
	CCA	31 (9)	13 (4)	4 (1)	10 (3)	4 (1)
	PCM	46 (13)	0 (0)	6 (2)	26 (7)	14 (4)
	PCS	8 (2)	2 (1)	1 (0)	2 (1)	3 (1)
**Cancer**						
	CCA	50 (14)	12 (3)	5 (1)	16 (5)	17 (5)
	PCM	1 (0)	0 (0)	0 (0)	1 (0)	0 (0)
	PCS	23 (7)	4 (1)	3 (1)	13 (4)	3 (1)
**Stroke**						
	CCA	15 (4)	1 (0)	1 (0)	3 (1)	10 (3)
	PCM	1 (0)	0 (0)	0 (0)	1 (0)	0 (0)
	PCS	5 (1)	0 (0)	0 (0)	3 (1)	2 (1)

^a^ More than 1 (person-centered) connected-care management activity possible.

Furthermore, our findings suggested that the most commonly used personalized ICT interventions involved telemonitoring or telemedicine systems (n=159) followed by Web-based applications on the Internet (n=117). In approximately one-fifth of the studies, the telephone (n=70) was used to connect patient and physician, mostly for consultation and education. For example, in the case of cardiovascular conditions, we found 18 studies on telephone intervention for connected care and in 19 studies on persons with diabetes, the telephone was used, often in combination with Internet-based interventions. In addition, the use of mobile phone apps ranked the highest in diabetes care activities (n=25).

The usage of social media, such as Twitter, was only incidentally mentioned in the reviewed studies, even though eHealth app and medical Internet-based interventions are paying increasing attention to social media [32]. A possible explanation could be that Twitter is less used in the relationship between a patient and his or her health care professional, which is the starting point for this scoping review, and more for accessing health information in general.

When comparing ICT-enabled PCC innovations used in different chronic diseases, several results stand out. First, in the case of cardiac patients, high-tech innovations connect remote monitoring software to implant devices (53%), such as pacemakers. Second, persons with a chronic stroke condition are beginning to use (serious) gaming and robot devices, specifically for rehabilitation purposes, which is a necessary treatment immediately after a stroke incident. Because technology is becoming smaller and cheaper, the possibilities of “wearable” smart technologies are increasing, and we expect to see more of these technologies in the future. Third, virtual clinics provide self-rehabilitation exercises. This technology combines a virtual clinic app on the Internet with telemonitoring systems.

The impact of PCC-ICT interventions on quality of life and health-related quality of life are positive (Table 9). Several studies claim an increase in quality of life (46/350, 13.1%) or health-related quality of life (28/350, 8.0%). It seems that enabling a person to manage his or her own disease through ICT leads to an improvement in the perceived quality of an individual’s daily life (quality of life) and an increase in the measurement of an individual’s well being affected over time by the disease, disability, or disorder (health-related quality of life).

The impact on cost and efficiency seems to be positive but less conclusive. Some studies reported positive impacts (38/350, 10.9%). Some of the studies, however, indicated negative impacts, either an increase in hospital (re)admission (6/350, 1.7%) or rise in costs (6/350, 1.7%). Our study suggests that not only could a person with a chronic disease benefit from an ICT-enabled PCC approach, but also that ICT-PCC yields organizational paybacks, although not in all cases. It could also lead, as was reported in some studies, to an increase in health care usage.

Other relevant study outcomes suggest that organizational barriers stand in the way of implementation of ICT-PCC, which is also supported by previous studies [26].

### Limitations

Although we covered a considerable number of studies, the search was limited to medical databases. Due to this system restriction, there is a chance that we have missed possible related articles in other domains, such as information systems research, social studies, and organizational change management research.

We realize that conducting a scoping study comes with limitations. We acknowledge the fact that the quantitative overview typical for scoping review results, unlike systematic reviews, does not appraise the quality of evidence in the primary research reports with a detailed analysis of a smaller and similar number of studies [22]. Because scoping reviews do not assess the quality of the studies, we included those studies in the review even though they did not reach the quality standard of some peer-reviewed journals. This also sets limitations on the results, which could only be described in general terms such as a “telemonitoring device.” Furthermore, we acknowledge the publication bias of a tendency to publish positive results that could yield a distorted overview of the scope of conducted research on ICT-enabled PCC. Lastly, our interpretations are limited to outcomes reported in the English language.

### Comparison With Prior Works

This scoping review mapped ICT-PCC interventions that are applied in chronic disease management to support patients to take an active part in their care and the decision-making process, and make it possible for patients to interact directly with health care providers and services about their personal health concerns. Our study distinguished 13 extracted care activities of connected care and PCC, which build on previous literature review studies on PCC and/or ICT, such as the one conducted by Aarts et al [17]. They extracted 2 care activities, namely the provision of support and education to patients and the promotion of mental health for patient-focused Internet interventions within the discipline of reproductive medicine.

Corresponding to our findings of less hospitalizations are the findings of the scoping review on the effects of PCC for patients with chronic heart failure in hospital settings. Ekman et al [9] found that a fully implemented PCC approach shortens hospital stays and maintains functional performance in patients hospitalized for worsening congestive heart failure without increasing the risk of readmission or jeopardizing patients’ health-related quality of life. However, Ekman’s study did not involve ICT and the focus was fully on the impact of a PCC approach. A comparable conclusion was drawn in a meta-analysis conducted on the outcomes of an Internet intervention and eHealth counseling on risk factors linked to certain chronic diseases [16].

### Theoretical Implication and Further Research Suggestions

This scoping research study has contributed to the growing scholarly interest in PCC and ICT interventions for self-management (of chronic conditions) by providing an overview of the extent and nature of the existing literature and evidence base involving the subset of ICT interventions in PCC for chronic conditions. Sixty relevant health studies have been identified regarding the big 5 chronic diseases to support patients and health care providers in the online and personalized management of these diseases.

For future research, we have 3 suggestions: first, given that hardly any of the studies showed a fully PCC-ICT approach, a logical next step is a qualitative study addressing the selection of the studies we found. Such a study can add qualitative insight and lead to placing an emphasis on building a framework. Second, given the 35 outcome indicators we identified, further research on the definition and measurement can help to further develop an evidence base for PCC and ICT for self-management of chronic disease. Third, we pose 2 challenging questions for further research:

1. How can ICT-enabled PCC be implemented in network organizations to support self-management of chronic patients in a person-centered care manner?
2. What does this mean for innovative care models?

### Practitioner and Managerial Implication

Concerning the impact of ICT-enabled PCC, this scoping review study found that empowerment (self-care) of the patient was the main outcome (15%) of the ICT interventions, followed by physical condition (14%), quality of life (13%), and health-related quality of life (8%). For the health care professional, the impact was highest when looking at clinical outcomes (14%). We also found a decrease in clinical outcomes in 2% of the studies. Regarding the most studied impact in the organization, we concluded that the outcome is less hospitalization (12%) and cost efficiency (11%). As far as the ICT intervention is concerned, the impact of feasibility (10%) is high. We also did find negative outcomes within the overall chronic disease categories: health-related quality of life decrease (5%), cost efficiency decrease (2%), and increase in hospitalization (2%).

### Conclusions

Hardly any of the interventions could be regarded as “fully” PCC meeting the 3 routines of initiating the partnership (patient narratives), working the partnership (shared decision making), and safeguarding the partnership (documenting the narrative). This review will be especially helpful to those deciding on areas where the further development of research or implementation of ICT for PCC may be warranted.

The scoping review investigated the extent, range, and nature of research activities regarding ICT interventions that have been studied to support patients and health care professionals in PCC management of the big 5 chronic diseases. From the initial 9380 search results, we identified 350 studies that qualified for inclusion. The largest share of ICT interventions studied sought to support patients in self-measurement of the body. The highest impact of ICT interventions (15%) of the studies on patients was measured on the increase of empowerment (self-care) closely followed by improvement in physical condition (14%), increase in quality of life (13%), health-related quality of life (8%), and self-efficacy (5%). Only 6% of the studies measured usability. This is disturbing since usability is an important fact for the acceptability of ICT by its users, and the lack of attention paid to usability in the reviewed studies indicates that there would be much to be gained from this.

The scoping review suggests that not only can persons with a chronic disease benefit from an ICT-enabled PCC approach, but also that ICT-PCC yields organizational paybacks, although not in all cases. It could also lead, as was reported in some studies, to an increase in health care usage. Other relevant study outcomes suggest that organizational barriers stand in the way of implementation of ICT-PCC, which is also supported by previous studies.

The impact of being connected to the health care professional by ICT is found to be the highest (12%) on a familiar clinical outcomes indicator versus a decrease in 2% of the studies. Remarkably, the most studied impact on organization outcome is not cost efficiency itself, but the related impact of less hospitalization (12%) closely followed by cost efficiency (11%).

Persons with a chronic disease are beginning to use (serious) gaming, social media, wearable technology, and robot devices for the management of diseases. Because technology overall is becoming smaller and cheaper, the possibilities of these smart technologies are increasing and we expect to see more of these technologies in the future.

## Multimedia Appendix 1

Overview of studies (n=350), qualified for inclusion.

## Abbreviations

CCA: connected-care activities
HRQL: health-related quality of life
ICT: information and communication technology
MeSH: Medical Subject Heading
PCC: person-centered care
PCM: person-centered self-measurement
PCS: person-centered shared management
PDF: portable document format
WHO: World Health Organization
