# Supplementary material for: Information and Communication Technology–Enabled Person-Centered Care for the “Big Five” Chronic Conditions: Scoping Review
Source: J Med Internet Res. 2015 Mar 27;17(3):e77. doi: 10.2196/jmir.3687 (PMC4393506; doi:10.2196/jmir.3687)
Supplement: Supplementary file 1 [file jmir_v17i3e77_app1.pdf]

Multimedia Appendix 1 Overview of studies (n=350), qualified for inclusion

Diabetes

1. Po YM. Telemedicine to improve patients' self-efficacy in managing diabetes. J Telemed Telecare. 2000;6(5):263-7. PubMed PMID: 11070586. Epub 2000/11/09.
2. Lahtela JT, Lamminen H. Telemedical devices in diabetes management. Ann Med. 2002;34(4):241-7. PubMed PMID: 12371708. Epub 2002/10/10.
3. Kerkenbush NL, Lasome CE. The emerging role of electronic diaries in the management of diabetes mellitus. AACN clinical issues. 2003 Aug;14(3):371-8. PubMed PMID: 12909805. Epub 2003/08/12.
4. Farmer A, Gibson OJ, Tarassenko L, Neil A. A systematic review of telemedicine interventions to support blood glucose self-monitoring in diabetes. Diabet Med. 2005 Oct;22(10):1372-8. PubMed PMID: 16176199. Epub 2005/09/24. eng.
5. Welch G, Shayne R. Interactive behavioral technologies and diabetes self-management support: Recent research findings from clinical trials. Current Diabetes Reports. 2006;6(2):130-6.
6. Brown LL, Lustria ML, Rankins J. A review of web-assisted interventions for diabetes management: maximizing the potential for improving health outcomes. J Diabetes Sci Technol. 2007 Nov;1(6):892-902. PubMed PMID: 19885163. Pubmed Central PMCID: Pmc2769687. Epub 2007/11/01.
7. Jaana M, Pare G. Home telemonitoring of patients with diabetes: A systematic assessment of observed effects. Journal of Evaluation in Clinical Practice. 2007;13(2):242-53.
8. Verhoeven F, Van Gemert-Pijnen L, Dijkstra K, Nijland N, Seydel E, Steehouder M. The contribution of teleconsultation and videoconferencing to diabetes care: A systematic literature review. Journal of Medical Internet Research. 2007;9(5):e37.
9. Dalton JE. Web-based care for adults with type 2 diabetes. Canadian Journal of Dietetic Practice and Research. 2008;69(4):185-91.
10. Krishna S, Boren SA. Diabetes self-management care via cell phone: a systematic review. J Diabetes Sci Technol. 2008 May;2(3):509-17. PubMed PMID: 19885219. Pubmed Central PMCID: Pmc2769746. Epub 2008/05/01.
11. Ontario HQ. Home telemonitoring for type 2 diabetes: an evidence-based analysis. Ont Health Technol Assess Ser. 2009;9(24):1-38. PubMed PMID: 23074529. Pubmed Central PMCID: Pmc3377533. Epub 2009/01/01.
12. Osborn CY, Mayberry LS, Mulvaney SA, Hess R. Patient web portals to improve diabetes outcomes: A systematic review. Current Diabetes Reports. 2010;10(6):422-35.
13. Samoocha D, Bruinvels DJ, Elbers NA, Anema JR, van der Beek AJ. Effectiveness of web-based interventions on patient empowerment: a systematic review and meta-analysis. J Med Internet Res. 2010;12(2):e23. PubMed PMID: 20581001. Pubmed Central PMCID: Pmc2956234. Epub 2010/06/29.
14. Verhoeven F, Tanja-Dijkstra K, Nijland N, Eysenbach G, van Gemert-Pijnen L. Asynchronous and synchronous teleconsultation for diabetes care: a systematic literature review. J Diabetes Sci Technol. 2010 May;4(3):666-84. PubMed PMID: 20513335. Pubmed Central PMCID: Pmc2901046. Epub 2010/06/02.
15. Mulvaney SA, Ritterband LM, Bosslet L. Mobile intervention design in diabetes: review and recommendations. Curr Diab Rep. 2011 Dec;11(6):486-93. PubMed PMID: 21960031. Epub 2011/10/01.

16. Salzsieder E, Augstein P. The Karlsburg Diabetes Management System: Translation from research to eHealth application. Journal of Diabetes Science and Technology. 2011;5(1):13-22.
17. Wei I, Pappas Y, Car J, Sheikh A, Majeed A. Computer-assisted versus oral-and-written dietary history taking for diabetes mellitus. Cochrane Database of Systematic Reviews [Internet]. 2011; (12). Available from: http://onlinelibrary.wiley.com/doi/10.1002/14651858.CD008488.pub2/abstract.
18. Holtz B, Lauckner C. Diabetes management via mobile phones: a systematic review. Telemed J E Health. 2012 Apr;18(3):175-84. PubMed PMID: 22356525. Epub 2012/02/24.
19. Siriwardena LS, Wickramasinghe WA, Perera KL, Marasinghe RB, Katulanda P, Hewapathirana R. A review of telemedicine interventions in diabetes care. J Telemed Telecare. 2012 Apr;18(3):164-8. PubMed PMID: 22362832. Epub 2012/03/01.
20. Fitzner K, Moss G. Telehealth - An effective delivery method for diabetes self-management education? Population Health Management. 2013;16(3):169-77.
21. Klonoff DC. The current status of mHealth for diabetes: will it be the next big thing? J Diabetes Sci Technol. 2013 May;7(3):749-58. PubMed PMID: 23759409. Epub 2013/06/14.
22. Marcolino MS, Maia JX, Alkmim MB, Boersma E, Ribeiro AL. Telemedicine application in the care of diabetes patients: systematic review and meta-analysis. PLoS One. 2013;8(11):e79246. PubMed PMID: 24250826. Pubmed Central PMCID: Pmc3826722. Epub 2013/11/20.
23. Pal K, Eastwood SV, Michie S, Farmer AJ, Barnard ML, Peacock R, et al. Computer-based diabetes self-management interventions for adults with type 2 diabetes mellitus. The Cochrane database of systematic reviews. 2013;3:CD008776.
24. Sieverdes JC, Treiber F, Jenkins C. Improving diabetes management with mobile health technology. American Journal of the Medical Sciences. 2013;345(4):289-95.
25. Biermann E, Dietrich W, Standl E. Telecare of diabetic patients with intensified insulin therapy. A randomized clinical trial. Studies in health technology and informatics. 2000;77:327-32. PubMed PMID: CN-00329493.
26. Glasgow RE, Toobert DJ. Brief, computer-assisted diabetes dietary self-management counseling: effects on behavior, physiologic outcomes, and quality of life. Medical care. 2000;38(11):1062-73.
27. Piette JD. Satisfaction with automated telephone disease management calls and its relationship to their use. The Diabetes educator. 2000;26(6):1003-10.
28. Piette JD, Weinberger M, McPhee SJ. The effect of automated calls with telephone nurse follow-up on patient-centered outcomes of diabetes care: a randomized, controlled trial. Medical care. 2000;38(2):218-30.
29. Smith L, Weinert C. Telecommunication support for rural women with diabetes. The Diabetes educator. 2000;26(4):645-55.
30. Bellazzi R, Larizza C, Montani S, Riva A, Stefanelli M, d'Annunzio G, et al. A telemedicine support for diabetes management: the T-IDDM project. Comput Methods Programs Biomed. 2002 Aug;69(2):147-61. PubMed PMID: 12100794. Epub 2002/07/09.
31. Biermann E, Dietrich W, Rihl J, Standl E. Are there time and cost savings by using telemanagement for patients on intensified insulin therapy?: A randomised, controlled trial. Computer Methods and Programs in Biomedicine. 2002;69(2):137-46.

32. Gomez EJ, Hernando ME, Garcia A, Del Pozo F, Cermeno J, Corcoy R, et al. Telemedicine as a tool for intensive management of diabetes: The DIABTel experience. Computer Methods and Programs in Biomedicine. 2002;69(2):163-77.
33. Howells L, Wilson AC, Skinner TC, Newton R, Morris AD, Greene SA. A randomized control trial of the effect of negotiated telephone support on glycaemic control in young people with Type 1 diabetes. Diabet Med. 2002 Aug;19(8):643-8. PubMed PMID: 12147144. Epub 2002/07/31.
34. Franklin V, Waller A, Pagliari C, Greene S. "Sweet Talk": text messaging support for intensive insulin therapy for young people with diabetes. Diabetes technology & therapeutics. 2003;5(6):991-6. PubMed PMID: 14709202. Epub 2004/01/08.
35. Izquierdo RE, Knudson PE, Meyer S, Kearns J, Ploutz-Snyder R, Weinstock RS. A comparison of diabetes education administered through telemedicine versus in person. Diabetes Care. 2003 Apr;26(4):1002-7. PubMed PMID: 12663564. Epub 2003/03/29.
36. Chan WM, Woo J, Hui E, Lau WW, Lai JC, Lee D. A community model for care of elderly people with diabetes via telemedicine. Appl Nurs Res. 2005 May;18(2):77-81. PubMed PMID: 15991104. Epub 2005/07/02.
37. Bond GE, Burr R, Wolf FM, Price M, McCurry SM, Teri L. Preliminary findings of the effects of comorbidities on a web-based intervention on self-reported blood sugar readings among adults age 60 and older with diabetes. Telemed J E Health. 2006 Dec;12(6):707-10. PubMed PMID: 17250494. Epub 2007/01/26.
38. Javaher P, Seidel G, Dierks ML. Participation in disease management of a health insurance company: Characteristics and assessment of the process and outcome parameters in the programme. Journal of Public Health. 2006;14(1):37-42.
39. Ladyzdynski P, Wojcicki JM, Krzymien J, Foltynski P, Migalska-Musial K, Tracz M, et al. Mobile telecare system for intensive insulin treatment and patient education. First applications for newly diagnosed type 1 diabetic patients. International Journal of Artificial Organs. 2006;29(11):1074-81.
40. Ma Y, Olendzki BC, Chiriboga D, Rosal M, Sinagra E, Crawford S, et al. PDA-assisted low glycemic index dietary intervention for type II diabetes: A pilot study. European Journal of Clinical Nutrition. 2006;60(10):1235-43.
41. Piette JD, Lange I, Issel M, Campos S, Bustamante C, Sapag J, et al. Use of telephone care in a cardiovascular disease management programme for type 2 diabetes patients in Santiago, Chile. Chronic Illness. 2006;2(2):87-96.
42. Benhamou PY, Melki V, Boizel R, Perreal F, Quesada JL, Bessieres-Lacombe S, et al. One-year efficacy and safety of Web-based follow-up using cellular phone in type 1 diabetic patients under insulin pump therapy: the PumpNet study. Diabetes and Metabolism. 2007;33(3):220-6.
43. Bond GE, Burr R, Wolf FM, Price M, McCurry SM, Teri L. The effects of a web-based intervention on the physical outcomes associated with diabetes among adults age 60 and older: A randomized trial. Diabetes Technology and Therapeutics. 2007;9(1):52-9.
44. Cadario F, Binotti M, Brustia M, Mercandino F, Moreno G, Esposito S, et al. Telecare for teenagers with type 1 diabetes: a trial. Minerva pediatrica. 2007 Aug;59(4):299-305. PubMed PMID: 17947836. Epub 2007/10/20.
45. Carroll AE, Marrero DG, Downs SM. The HealthPia GlucoPack Diabetes phone: a usability study. Diabetes technology & therapeutics. 2007 Apr;9(2):158-64. PubMed PMID: 17425441. Epub 2007/04/12.

46. Kim HS. A randomized controlled trial of a nurse short-message service by cellular phone for people with diabetes. International Journal of Nursing Studies. 2007;44(5):687-92.
47. Kim HS, Jeong HS. A nurse short message service by cellular phone in type-2 diabetic patients for six months. J Clin Nurs. 2007 Jun;16(6):1082-7. PubMed PMID: 17518883. Epub 2007/05/24.
48. Kollmann A, Riedl M, Kastner P, Schreier G, Ludvik B. Feasibility of a mobile phone-based data service for functional insulin treatment of type 1 diabetes mellitus patients. Journal of Medical Internet Research. 2007;9(5):e36.
49. Buckley K, Tran B, Agazio J, Wuertz E. A community-based telehealth programme for elderly low-income African Americans. Journal on Information Technology in Healthcare. 2008;6(6):400-12.
50. Faridi Z, Liberti L, Shuval K, Northrup V, Ali A, Katz DL. Evaluating the impact of mobile telephone technology on type 2 diabetic patients' self-management: The NICHE pilot study. Journal of Evaluation in Clinical Practice. 2008;14(3):465-9.
51. Forjuoh SN, Reis MD, Couchman GR, Ory MG. Improving diabetes self-care with a PDA in ambulatory care. Telemedicine and e-Health. 2008;14(3):273-9.
52. Handley MA, Shumway M, Schillinger D. Cost-effectiveness of automated telephone self-management support with nurse care management among patients with diabetes. Annals of Family Medicine. 2008;6(6):512-8.
53. Quinn CC, Clough SS, Minor JM, Lender D, Okafor MC, Gruber-Baldini A. WellDoc mobile diabetes management randomized controlled trial: change in clinical and behavioral outcomes and patient and physician satisfaction. Diabetes technology & therapeutics. 2008 Jun;10(3):160-8. PubMed PMID: 18473689. Epub 2008/05/14.
54. Sevick MA, Zickmund S, Korytkowski M, Piraino B, Sereika S, Mihalko S, et al. Design, feasibility, and acceptability of an intervention using personal digital assistant-based self-monitoring in managing type 2 diabetes. Contemporary Clinical Trials. 2008;29(3):396-409.
55. Trief PM, Sandberg J, Izquierdo R, Morin PC, Shea S, Brittain R, et al. Diabetes management assisted by telemedicine: Patient perspectives. Telemedicine and e-Health. 2008;14(7):647-55.
56. Wagnild G, MacCart JG, Mitchell S, Tyabah K, Leenknecht C, Meszaros JF. A telecommunications intervention for frontier patients with diabetes. Telemedicine and e-Health. 2008;14(8):793-800.
57. Albisser AM, Alejandro R, Sperlich M, Ricordi C. Closing the circle of care with new firmware for diabetes: MyDiaBase+RxChecker. Journal of diabetes science and technology. 2009;3(3):619-23.
58. Boaz M, Hellman K, Wainstein J. An automated telemedicine system improves patient-reported well-being. Diabetes Technology and Therapeutics. 2009;11(3):181-6.
59. Derose SF, Nakahiro RK, Ziel FH. Automated messaging to improve compliance with diabetes test monitoring. American Journal of Managed Care. 2009;15(7):425-31.
60. Jennings A, Powell J, Armstrong N, Sturt J, Dale J. A virtual clinic for diabetes self-management: pilot study. Journal of medical Internet research. 2009;11(1):e10.
61. Quinn CC, Gruber-Baldini AL, Shardell M, Weed K, Clough SS, Peeples M, et al. Mobile diabetes intervention study: Testing a personalized treatment/behavioral communication intervention for blood glucose control. Contemporary Clinical Trials. 2009;30(4):334-46.

62. Roek MG, Welschen LM, Kostense PJ, Dekker JM, Snoek FJ, Nijpels G. Web-based guided insulin self-titration in patients with type 2 diabetes: The Di@log study. Design of a cluster randomised controlled trial [TC1316]. BMC Family Practice. 2009;10.
63. Shea S, Weinstock RS, Teresi JA, Palmas W, Starren J, Cimino JJ, et al. A randomized trial comparing telemedicine case management with usual care in older, ethnically diverse, medically underserved patients with diabetes mellitus: 5 year results of the IDEATel study. J Am Med Inform Assoc. 2009 Jul-Aug;16(4):446-56. PubMed PMID: 19390093. Pubmed Central PMCID: Pmc2705246. Epub 2009/04/25.
64. Trief PM, Teresi JA, Eimicke JP, Shea S, Weinstock RS. Improvement in diabetes self-efficacy and glycaemic control using telemedicine in a sample of older, ethnically diverse individuals who have diabetes: the IDEATel project. Age Ageing. 2009 Mar;38(2):219-25. PubMed PMID: 19171951. Epub 2009/01/28.
65. Glasgow RE, Kurz D, King D, Dickman JM, Faber AJ, Halterman E, et al. Outcomes of minimal and moderate support versions of an internet-based diabetes self-management support program. Journal of General Internal Medicine. 2010;25(12):1315-22.
66. Lehmkuhl HD, Storch EA, Cammarata C, Meyer K, Rahman O, Silverstein J, et al. Telehealth behavior therapy for the management of type 1 diabetes in adolescents. J Diabetes Sci Technol. 2010 Jan;4(1):199-208. PubMed PMID: 20167185. Pubmed Central PMCID: Pmc2825642. Epub 2010/02/20.
67. Rossi MCE, Nicolucci A, Di Bartolo P, Bruttomesso D, Girelli A, Ampudia FJ, et al. Diabetes interactive diary: A new telemedicine system enabling flexible diet and insulin therapy while improving quality of life: An open-label, international, multicenter, randomized study. Diabetes Care. 2010;33(1):109-15.
68. Vargas-Lombardo M, Jipsion A, Alvarez H, Ruiz EM, Mora EV. Scope of information communications technology in the health of diabetes patients in poor rural zones of panama through holistic, interactive, and persuasive model to facilitate self-care of diabetes patients. Diabetes Technology and Therapeutics. 2010;12(9):717-22.
69. Bujnowska-Fedak MM, Puchala E, Steciwko A. The impact of telehome care on health status and quality of life among patients with diabetes in a primary care setting in Poland. Telemedicine journal and e-health : the official journal of the American Telemedicine Association. 2011;17(3):153-63.
70. Carroll AE, DiMeglio LA, Stein S, Marrero DG. Using a cell phone-based glucose monitoring system for adolescent diabetes management. Diabetes Educ. 2011 Jan-Feb;37(1):59-66. PubMed PMID: 21106908. Epub 2010/11/26.
71. Charpentier G, Benhamou PY, Dardari D, Clergeot A, Franc S, Schaepelynck-Belicar P, et al. The diabeo software enabling individualized insulin dose adjustments combined with telemedicine support improves HbA1c in poorly controlled type 1 diabetic patients: A 6-month, randomized, open-label, parallel-group, multicenter trial (TeleDiab 1 study). Diabetes Care. 2011;34(3):233-9.
72. Chen SY, Chang YH, Hsu HC, Lee YJ, Hung YJ, Hsieh CH. One-year efficacy and safety of the telehealth system in poorly controlled type 2 diabetic patients receiving insulin therapy. Telemed J E Health. 2011 Nov;17(9):683-7. PubMed PMID: 21882998. Epub 2011/09/03.
73. Cho JH, Kwon HS, Kim HS, Oh JA, Yoon KH. Effects on diabetes management of a health-care provider mediated, remote coaching system via a PDA-type glucometer and the Internet. J Telemed Telecare. 2011;17(7):365-70. PubMed PMID: 21933896. Epub 2011/09/22.

74. Ciemins E, Coon P, Peck R, Holloway B, Min SJ. Using telehealth to provide diabetes care to patients in rural Montana: findings from the promoting realistic individual self-management program. Telemed J E Health. 2011 Oct;17(8):596-602. PubMed PMID: 21859347. Pubmed Central PMCID: Pmc3208251. Epub 2011/08/24.
75. Glasgow RE, Christiansen SM, Kurz D, King DK, Woolley T, Faber AJ, et al. Engagement in a diabetes self-management website: usage patterns and generalizability of program use. J Med Internet Res. 2011;13(1):e9. PubMed PMID: 21371992. Pubmed Central PMCID: Pmc3221359. Epub 2011/03/05.
76. Jordan RE, Lancashire RJ, Adab P. An evaluation of Birmingham Own Health telephone care management service among patients with poorly controlled diabetes. A retrospective comparison with the General Practice Research Database. BMC Public Health. 2011;11:707. PubMed PMID: 21929804. Pubmed Central PMCID: Pmc3191515. Epub 2011/09/21.
77. Kulnawan N, Jiamjarasrangsi W, Suwanwalaikorn S, Kittisopee T, Meksawan K, Thadpitakkul N, et al. Development of diabetes telephone-linked care system for self-management support and acceptability test among type 2 diabetic patients. Journal of the Medical Association of Thailand. 2011;94(10):1189-97.
78. Ladyzynski P, Foltynski P, Molik M, Tarwacka J, Migalska-Musial K, Mlynarczuk M, et al. Area of the diabetic ulcers estimated applying a foot scanner-based home telecare system and three reference methods. Diabetes Technology and Therapeutics. 2011;13(11):1101-7.
79. Nijland N, van Gemert-Pijnen JE, Kelders SM, Brandenburg BJ, Seydel ER. Factors influencing the use of a Web-based application for supporting the self-care of patients with type 2 diabetes: a longitudinal study. J Med Internet Res. 2011;13(3):e71. PubMed PMID: 21959968. Pubmed Central PMCID: Pmc3222177. Epub 2011/10/01.
80. O'Shea T, McSorley J, Hardy T, Pereira P, Vijayaraghavan S. Web-based outpatient consultations in diabetes. Diabetic Medicine. 2011;28:88-9.
81. Pelletier AC, Jethwani K, Bello H, Kvedar J, Grant RW. Implementing a web-based home monitoring system within an academic health care network: Barriers and facilitators to innovation diffusion. Journal of Diabetes Science and Technology. 2011;5(1):32-8.
82. Quinn CC, Shardell MD, Terrin ML, Barr EA, Ballew SH, Gruber-Baldini AL. Cluster-randomized trial of a mobile phone personalized behavioral intervention for blood glucose control. Diabetes Care. 2011;34(9):1934-42.
83. White S. Telemonitoring in the renal program: Empowering patients and engaging providers. Peritoneal Dialysis International. 2011;31:S29.
84. Bell AM, Fonda SJ, Walker MS, Schmidt V, Vigersky RA. Mobile phone-based video messages for diabetes self-care support. J Diabetes Sci Technol. 2012 Mar;6(2):310-9. PubMed PMID: 22538140. Pubmed Central PMCID: Pmc3380772. Epub 2012/04/28. eng.
85. Bursell SE, Brazionis L, Jenkins A. Telemedicine and ocular health in diabetes mellitus. Clinical and Experimental Optometry. 2012;95(3):311-27.
86. Goodarzi M, Ebrahimzadeh I, Rabi A, Saedipoor B, Jafarabadi MA. Impact of distance education via mobile phone text messaging on knowledge, attitude, practice and self efficacy of patients with type 2 diabetes mellitus in Iran. Journal of Diabetes and Metabolic Disorders. 2012;11(1):1-8.
87. Rossi MC, Nicolucci A, Lucisano G, Di Bartolo P, Miselli V, Anichini R, et al. nullDiabetes Interactive Diarynull Telemedicine System vs. Standard Carbohydrate

Counting Education in Type 1 Diabetes: Results of a randomized trial. Diabetes. 2012;61:A292.
88. Tenforde M, Nowacki A, Jain A, Hickner J. The association between personal health record use and diabetes quality measures. J Gen Intern Med. 2012 Apr;27(4):420-4. PubMed PMID: 22005937. Pubmed Central PMCID: Pmc3304034. Epub 2011/10/19.
89. Williams ED, Bird D, Forbes AW, Russell A, Ash S, Friedman R, et al. Randomised controlled trial of an automated, interactive telephone intervention (TLC Diabetes) to improve type 2 diabetes management: baseline findings and six-month outcomes. BMC public health. 2012;12:602.
90. Zolfaghari M, Mousavifar SA, Haghani H. Mobile phone text messaging and Telephone follow-up in type 2 diabetic patients for 3 months: A comparative study. Journal of Diabetes and Metabolic Disorders. 2012;11(1).
91. Alkadi K, Roudsari A. TeMaD system: telecare for managing diabetes in Saudi Arabia. Stud Health Technol Inform. 2013;183:57-62. PubMed PMID: 23388255. Epub 2013/02/08.
92. Arora S, Burner E, Lam J, De Santos R, Meeks A, Menchine M. Trial to examine text-message based mhealth in ed patients with diabetes (TExT-MED). Academic Emergency Medicine. 2013;20(5):S180.
93. Blackberry ID, Furler JS, Best JD, Chondros P, Vale M, Walker C, et al. Effectiveness of general practice based, practice nurse led telephone coaching on glycaemic control of type 2 diabetes: The Patient Engagement and Coaching for Health (PEACH) pragmatic cluster randomised controlled trial. BMJ (Online). 2013;347(7926).
94. Chen L, Chuang LM, Chang CH, Wang CS, Wang IC, Chung Y, et al. Evaluating self-management behaviors of diabetic patients in a telehealthcare program: longitudinal study over 18 months. J Med Internet Res. 2013;15(12):e266. PubMed PMID: 24323283. Epub 2013/12/11.
95. Glasgow RE, Strycker LA, King DK, Toobert DJ. Understanding Who Benefits at Each Step in an Internet-Based Diabetes Self-Management Program: Application of a Recursive Partitioning Approach. Med Decis Making. 2013 Aug 1. PubMed PMID: 23913917. Epub 2013/08/06.
96. Kirwan M, Vandelanotte C, Fenning A, Duncan MJ. Diabetes self-management smartphone application for adults with type 1 diabetes: randomized controlled trial. J Med Internet Res. 2013;15(11):e235. PubMed PMID: 24225149. Pubmed Central PMCID: Pmc3841374. Epub 2013/11/15.
97. Leichter SB, Bowman K, Adkins RA, Jelsovsky Z. Impact of remote management of diabetes via computer: the 360 study--a proof-of-concept randomized trial. Diabetes technology & therapeutics. 2013 May;15(5):434-8. PubMed PMID: 23537419. Epub 2013/03/30.
98. Louch G, Dalkin S, Bodansky J, Conner M. An exploratory randomised controlled trial using short messaging service to facilitate insulin administration in young adults with type 1 diabetes. Psychology, health & medicine. 2013;18(2):166-74. PubMed PMID: 22646659. Epub 2012/06/01.
99. Osborn CY, Mayberry LS, Wallston KA, Johnson KB, Elasy TA. Understanding patient portal use: implications for medication management. J Med Internet Res. 2013;15(7):e133. PubMed PMID: 23823974. Pubmed Central PMCID: Pmc3713921. Epub 2013/07/05.
100. Rossi MC, Nicolucci A, Lucisano G, Pellegrini F, Di Bartolo P, Miselli V, et al. Impact of the "diabetes interactive diary" telemedicine system on metabolic control, risk of

hypoglycemia, and quality of life: A randomized clinical trial in type 1 diabetes. Diabetes Technology and Therapeutics. 2013;15(8):670-9.
101. Thornby KA, Edquist N. Diabetes apps: Impacting patients' lives. 2013.
102. Wade-Vuturo AE, Mayberry LS, Osborn CY. Secure messaging and diabetes management: experiences and perspectives of patient portal users. J Am Med Inform Assoc. 2013 May 1;20(3):519-25. PubMed PMID: 23242764. Pubmed Central PMCID: Pmc3628058. Epub 2012/12/18.
103. Weymann N, Harter M, Petrak F, Dirmaier J. Health information, behavior change, and decision support for patients with type 2 diabetes: Development of a tailored, preference-sensitive health communication application. Patient Preference and Adherence. 2013;7:1091-9.

Chronic cardiovascular diseases

1. Lusignan S, Meredith K, Wells S, Leatham E, Johnson P. A controlled pilot study in the use of telemedicine in the community on the management of heart failure--a report of the first three months. Studies in health technology and informatics. 1999;64:126-37. PubMed PMID: CN-00371805.
2. Ades PA, Pashkow FJ, Fletcher G, Pina IL, Zohman LR, Nestor JR. A controlled trial of cardiac rehabilitation in the home setting using electrocardiographic and voice transtelephonic monitoring. American heart journal. 2000;139(3):543-8. PubMed PMID: CN-00275537.
3. Bennett SJ, Hays LM, Embree JL, Arnould M. Heart Messages: a tailored message intervention for improving heart failure outcomes. J Cardiovasc Nurs. 2000 Jul;14(4):94-105. PubMed PMID: 10902106. Epub 2000/07/21.
4. Creason H. Congestive heart failure telemanagement clinic. Lippincotts Case Manag. 2001 Jul-Aug;6(4):146-56. PubMed PMID: 16398064. Epub 2006/01/10. eng.
5. de Lusignan S, Wells S, Johnson P, Meredith K, Leatham E. Compliance and effectiveness of 1 year's home telemonitoring. The report of a pilot study of patients with chronic heart failure. Eur J Heart Fail. 2001 Dec;3(6):723-30. PubMed PMID: 11738225. Epub 2001/12/12.
6. Lusignan S, Wells S, Johnson P, Meredith K, Leatham E. Compliance and effectiveness of 1 year's home telemonitoring. The report of a pilot study of patients with chronic heart failure. European journal of heart failure. 2001;3(6):723-30. PubMed PMID: CN-00376109.
7. Artinian NT, Harden JK, Kronenberg MW, Vander Wal JS, Daher E, Stephens Q, et al. Pilot study of a Web-based compliance monitoring device for patients with congestive heart failure. Heart and Lung: Journal of Acute and Critical Care. 2003;32(4):226-33.
8. Stroetmann KA, Stroetmann VN, Westerteicher C. Implementation of TeleCare services: benefit assessment and organisational models. Stud Health Technol Inform. 2003;97:131-41. PubMed PMID: 15537237. Epub 2004/11/13.
9. Ross SE, Moore LA, Earnest MA, Wittevrongel L, Lin CT. Providing a web-based online medical record with electronic communication capabilities to patients with congestive heart failure: randomized trial. J Med Internet Res. 2004 May 14;6(2):e12. PubMed PMID: 15249261. Pubmed Central PMCID: Pmc1550594. Epub 2004/07/14.

10. Roth A, Kajiloti I, Elkayam I, Sander J, Kehati M, Golovner M. Telecardiology for patients with chronic heart failure: The 'SHL' experience in Israel. International Journal of Cardiology. 2004;97(1):49-55.
11. Schneider NM. Managing congestive heart failure using home telehealth. Home Healthc Nurse. 2004 Oct;22(10):719-22. PubMed PMID: 15486513. Epub 2004/10/16.
12. Wong BM, Yung BM, Wong A, Chow CM, Abramson BL. Increasing internet use among cardiovascular patients: New opportunies for heart health promotion. Canadian Journal of Cardiology. 2005;21(4):349-54.
13. Gottlieb S, Blum K. Coordinated care, telemonitoring, and the therapeutic relationship: Heart failure management in the United States. Disease Management and Health Outcomes. 2006;14(SUPPL. 1):29-31.
14. Kashem A, Droogan MT, Santamore WP, Wald JW, Marble JF, Cross RC, et al. Web-based internet telemedicine management of patients with heart failure. Telemedicine Journal and e-Health. 2006;12(4):439-47.
15. Quinn C. Low-technology heart failure care in home health: improving patient outcomes. Home Healthc Nurse. 2006 Sep;24(8):533-40. PubMed PMID: 17012959. Epub 2006/10/03.
16. Riegel B, Carlson B, Glaser D, Romero T. Randomized Controlled Trial of Telephone Case Management in Hispanics of Mexican Origin With Heart Failure. Journal of Cardiac Failure. 2006;12(3):211-9.
17. Roth A, Korb H, Gadot R, Kalter E. Telecardiology for patients with acute or chronic cardiac complaints: The 'SHL' experience in Israel and Germany. International Journal of Medical Informatics. 2006;75(9):643-5.
18. Clark RA, Yallop JJ, Piterman L, Croucher J, Tonkin A, Stewart S, et al. Adherence, adaptation and acceptance of elderly chronic heart failure patients to receiving healthcare via telephone-monitoring. European Journal of Heart Failure. 2007;9(11):1104-11.
19. Ramachandran K, Husain N, Maikhuri R, Seth S, Vij A, Kumar M, et al. Impact of a comprehensive telephone-based disease management programme on quality-of-life in patients with heart failure. National Medical Journal of India. 2007;20(2):67-73.
20. Zutz A, Ignaszewski A, Bates J, Lear SA. Utilization of the internet to deliver cardiac rehabilitation at a distance: a pilot study. Telemed J E Health. 2007 Jun;13(3):323-30. PubMed PMID: 17603835. Epub 2007/07/03.
21. Antonicelli R, Testarmata P, Spazzafumo L, Gagliardi C, Bilo G, Valentini M, et al. Impact of telemonitoring at home on the management of elderly patients with congestive heart failure. Journal of telemedicine and telecare. 2008;14(6):300-5.
22. Bai VT, Srivatsa SK. Portable telecardiac system for arrhythmia monitoring and alerting. International Journal of Healthcare Technology and Management. 2008;9(5-6):517-25.
23. Balk AH, Davidse W, Dommelen P, Klaassen E, Caliskan K, van der Burgh P, et al. Tele-guidance of chronic heart failure patients enhances knowledge about the disease. A multi-centre, randomised controlled study. Eur J Heart Fail. 2008 Nov;10(11):1136-42. PubMed PMID: 18790668. Epub 2008/09/16.
24. Boriani G, Diemberger I, Martignani C, Biffi M, Valzania C, Bertini M, et al. Telecardiology and remote monitoring of implanted electrical devices: the potential for fresh clinical care perspectives. J Gen Intern Med. 2008 Jan;23 Suppl 1:73-7. PubMed PMID: 18095049. Pubmed Central PMCID: Pmc2150639. Epub 2008/01/10.
25. Dansky KH, Vasey J, Bowles K. Impact of telehealth on clinical outcomes in patients with heart failure. Clinical Nursing Research. 2008;17(3):182-99.

26. Dansky KH, Vasey J, Bowles K. Use of telehealth by older adults to manage heart failure. Research in gerontological nursing. 2008;1(1):25-32.
27. Katalinic A, Waldmann A, Schwaab B, Richardt G, Sheikhzadeh A, Raspe H. The TeleGuard trial of additional telemedicine care in CAD patients. 1 Utilization of the system. J Telemed Telecare. 2008;14(1):17-21. PubMed PMID: 18318924. Epub 2008/03/06. eng.
28. Masella C, Zanaboni P, Di Stasi F, Gilardi S, Ponzi P, Valsecchi S. Assessment of a remote monitoring system for implantable cardioverter defibrillators. Journal of telemedicine and telecare. 2008;14(6):290-4.
29. Schwarz KA, Mion LC, Hudock D, Litman G. Telemonitoring of heart failure patients and their caregivers: a pilot randomized controlled trial. Progress in cardiovascular nursing. 2008;23(1):18-26.
30. Woodend AK, Sherrard H, Fraser M, Stuewe L, Cheung T, Struthers C. Telehome monitoring in patients with cardiac disease who are at high risk of readmission. Heart and Lung: Journal of Acute and Critical Care. 2008;37(1):36-45.
31. Bernard ML, Quin EM, Gold MR. Remote Monitoring-The Future of Implantable Cardioverter-Defibrillator Follow-up. Cardiac Electrophysiology Clinics. 2009;1(1):193-200.
32. Dar O, Riley J, Chapman C, Dubrey SW, Morris S, Rosen SD, et al. A randomized trial of home telemonitoring in a typical elderly heart failure population in North West London: Results of the Home-HF study. European Journal of Heart Failure. 2009;11(3):319-25.
33. Mortara A, Pinna GD, Johnson P, Maestri R, Capomolla S, La Rovere MT, et al. Home telemonitoring in heart failure patients: The HHH study (Home or Hospital in Heart Failure). European Journal of Heart Failure. 2009;11(3):312-8.
34. Ramaekers BLT, Janssen-Boyne JJ, Gorgels APM, Vrijhoef HJM. Adherence among telemonitored patients with heart failure to pharmacological and nonpharmacological recommendations. Telemedicine and e-Health. 2009;15(6):517-24.
35. Roth A, Malov N, Steinberg DM, Yanay Y, Elizur M, Tamari M, et al. Telemedicine for post-myocardial infarction patients: An observational study. Telemedicine and e-Health. 2009;15(1):24-30.
36. Whitten P, Bergman A, Meese MA, Bridwell K, Jule K. St. Vincent's Home telehealth for congestive heart failure patients. Telemed J E Health. 2009 Mar;15(2):148-53. PubMed PMID: 19292623. Epub 2009/03/19.
37. Finkelstein J, Cha E, Dennison CR. Exploring feasibility of home telemanagement in African Americans with congestive heart failure. Stud Health Technol Inform. 2010;160(Pt 1):535-9. PubMed PMID: 20841744. Epub 2010/09/16.
38. Gonzalez B, Domingo M, Lupon J, Lopez R, Ramos A, Crespo E, et al. Use of telemedicine (Motiva-Philips) in an heart failure unit: Changes in patient's behaviour and impact on quality of life: The CARME study (CAtalan Remote Management Evaluation). European Journal of Heart Failure, Supplement. 2010;9:S108.
39. Hannah J, Humphrey G, Doughty R, McGrinder H, Bos N, Bowman C. Telehealth in heart failure management: A proof of principle study. Heart Lung and Circulation. 2010;19:S13-S4.
40. Helms TM, Pelleter J, Kroettinger A, Budych K, Thoden E, Sohn S, et al. Advantages and restrictions of telemonitoring in chronic heart failure - Experiences and results from the integrated care concept Telemedicine for the heart. European Heart Journal. 2010;31:226-7.

41. Janssen H. Longterm cost-effectiveness analysis of a telemedicine programme for patients with chronic heart failure. European Heart Journal. 2010;31:226.
42. Knotter N, Meregalli PG, Kok WE, De Voogt WG, De Beurs M, Cherpanath-Paes C, et al. Home telemonitoring in congestive heart failure: Patients perspective. European Journal of Heart Failure, Supplement. 2010;9:S51.
43. Kulshreshtha A, Kvedar JC, Goyal A, Halpern EF, Watson AJ. Use of remote monitoring to improve outcomes in patients with heart failure: A pilot trial. International Journal of Telemedicine and Applications. 2010.
44. Lobodzinski SS, Jadalla AA. Integrated heart failure telemonitoring system for homecare. Cardiol J. 2010;17(2):200-4. PubMed PMID: 20544625. Epub 2010/06/15. eng.
45. Maric B, Kaan A, Araki Y, Ignaszewski A, Lear SA. The use of the Internet to remotely monitor patients with heart failure. Telemedicine journal and e-health : the official journal of the American Telemedicine Association. 2010;16(1):26-33.
46. Seto E, Leonard KJ, Masino C, Cafazzo JA, Barnsley J, Ross HJ. Attitudes of heart failure patients and health care providers towards mobile phone-based remote monitoring. Journal of medical Internet research. 2010;12(4):e55.
47. Zugck C, Cebola R, Frankenstein L, Nelles M, Taeger T, Pribe R, et al. Telemedicine reduces hospitalisation rates in patients with chronic heart failure-results of the randomized HiTel trial. European Journal of Heart Failure, Supplement. 2010;9:S170.
48. Ando K, Koyama J, Abe Y, Sato T, Shoda M, Soga Y, et al. Feasibility evaluation of a remote monitoring system for implantable cardiac devices in Japan. International heart journal. 2011;52(1):39-43. PubMed PMID: 21321467. Epub 2011/02/16.
49. Baker DW, Dewalt DA, Schillinger D, Hawk V, Ruo B, Bibbins-Domingo K, et al. The effect of progressive, reinforcing telephone education and counseling versus brief educational intervention on knowledge, self-care behaviors and heart failure symptoms. Journal of Cardiac Failure. 2011;17(10):789-96.
50. Bergmann MW, Kuck KH, Krenz I. Volume regulation in cardiac insufficiency: Possibilities of telephone coaching and peritoneal dialysis. Herz. 2011;36(7):614-9.
51. Gupta A, Kashem A, Gonzalez J, Alkhouli M, Homko C, Santimore W, et al. The utility of specific measure for heart failure patient in web-based management: Reliability of kansas city cardiomyopathy questionnaire. Circulation: Cardiovascular Quality and Outcomes. 2011;4(6).
52. Jarvis-Selinger S, Bates J, Araki Y, Lear SA. Internet-based support for cardiovascular disease management. International Journal of Telemedicine and Applications. 2011.
53. Metten L, Zucca F, Haver Y, Neukirch B, Rauchhaus M. Effects of intensified care for heart failure patients by telemonitoring. European Journal of Cardiovascular Nursing. 2011;10:S26.
54. Seto E, Leonard KJ, Cafazzo JA, Masino C, Barnsley J, Ross HJ. Mobile phone-based remote patient monitoring improves heart failure management and outcomes: A randomized controlled trial. Journal of the American College of Cardiology. 2011;57(14):E1260.
55. Acosta-Lobos A, Riley JP, Cowie MR. Current and future technologies for remote monitoring in cardiology and evidence from trial data. Future Cardiology. 2012;8(3):425-37.
56. Al Khateeb M. Telephone intervention in chronic heart failure: Quality project. Journal of the Saudi Heart Association. 2012;24(4):289.

57. Blasco A, Carmona M, Fernandez-Lozano I, Salvador CH, Pascual M, Sagredo PG, et al. Evaluation of a telemedicine service for the secondary prevention of coronary artery disease. J Cardiopulm Rehabil Prev. 2012 Jan-Feb;32(1):25-31. PubMed PMID: 22113368. Epub 2011/11/25.
58. Bohme S, Geiser C, Muhlenhoff T, Holtmann J, Renneberg B. Telephone counseling for patients with chronic heart failure: results of an evaluation study. Int J Behav Med. 2012 Sep;19(3):288-97. PubMed PMID: 21732211. Epub 2011/07/07.
59. Karanam C, Dayanand S, Dang S, Cobian S, Gomez-Marin O, Mallon S, et al. Outcomes from a mobile-phone study for heart failure in an ethnically diverse County Hospital. Journal of the American Geriatrics Society. 2012;60:S221.
60. Landolina M, Perego GB, Lunati M, Curnis A, Guenzati G, Vicentini A, et al. Remote monitoring reduces healthcare use and improves quality of care in heart failure patients with implantable defibrillators: the evolution of management strategies of heart failure patients with implantable defibrillators (EVOLVO) study. Circulation. 2012 Jun 19;125(24):2985-92. PubMed PMID: 22626743. Epub 2012/05/26.
61. Seto E, Leonard KJ, Cafazzo JA, Barnsley J, Masino C, Ross HJ. Mobile phone-based telemonitoring for heart failure management: a randomized controlled trial. J Med Internet Res. 2012;14(1):e31. PubMed PMID: 22356799. Pubmed Central PMCID: Pmc3374537. Epub 2012/02/24.
62. Zanaboni P, Marzegalli M, Landolina ME, Lunati M, Perego GB, Guenzati G, et al. Evaluation of telemonitoring for heart failure patients with implantable defibrillators: The evolvo (evolution of management strategies of heart failure patients with implantable defibrillators) study. Value in Health. 2012;15(7):A281.
63. Gupta J, Fletcher T, Larcombe T, Gupta T. Telemonitoring as bridge to discharge in advanced heart failure. European Journal of Heart Failure. 2013;12:S296.
64. Lambrinou E, Kalogirou F, Protopapas A, Papathanassoglou E, Barberis V, Sourtzi P, et al. Management of patients with heart failure using education or education & yelephone or telephone in Cyprus (MEETTinCy) trial. Preliminary results. European Journal of Heart Failure. 2013;12:S220.
65. Sabatier R, Coutance G, Belin A, Biannic C, Loiselet P, Pradere G, et al. Three months educational remote telemonitoring in elderly patients with heart failure reduces hospitalizations for acute heart failure at one year: A randomized trial. European Journal of Heart Failure. 2013;12:S313.
66. Zanaboni P, Landolina M, Marzegalli M, Lunati M, Perego GB, Guenzati G, et al. Cost-utility analysis of the EVOLVO study on remote monitoring for heart failure patients with implantable defibrillators: randomized controlled trial. J Med Internet Res. 2013;15(5):e106. PubMed PMID: 23722666. Pubmed Central PMCID: Pmc3670725. Epub 2013/06/01.
67. Scalvini S, Giordano A. Management of patients with heart failure: The role of Telecare. Monaldi Archives for Chest Disease. 2002;58(3):252-5.
68. Louis AA, Turner T, Gretton M, Baksh A, Cleland JG. A systematic review of telemonitoring for the management of heart failure. Eur J Heart Fail. 2003 Oct;5(5):583-90. PubMed PMID: 14607195. Epub 2003/11/11.
69. Jerant AF, Nesbitt TS. Heart failure disease management incorporating telemedicine: A critical review. Journal of Clinical Outcomes Management. 2005;12(4):207-17.
70. Martinez A, Everss E, Rojo-Alvarez JL, Figal DP, Garcia-Alberola A. A systematic review of the literature on home monitoring for patients with heart failure. J Telemed Telecare. 2006;12(5):234-41. PubMed PMID: 16848935. Epub 2006/07/20.

71. Clark RA, Inglis SC, McAlister FA, Cleland JGF, Stewart S. Telemonitoring or structured telephone support programmes for patients with chronic heart failure: Systematic review and meta-analysis. British Medical Journal. 2007;334(7600):942-5.
72. Inglis SC, Clark RA, Cleland JGF, McAlister F, Stewart S. Structured telephone support or telemonitoring programs for patients with chronic heart failure. Cochrane Database of Systematic Reviews. 2008 (3).
73. Dang S, Dimmick S, Kelkar G. Evaluating the evidence base for the use of home telehealth remote monitoring in elderly with heart failure. Telemed J E Health. 2009 Oct;15(8):783-96. PubMed PMID: 19831704. Epub 2009/10/17.
74. Maric B, Kaan A, Ignaszewski A, Lear SA. A systematic review of telemonitoring technologies in heart failure. European Journal of Heart Failure. 2009;11(5):506-17.
75. Riley JP, Cowie MR. Telemonitoring in heart failure. Heart. 2009 Dec;95(23):1964-8. PubMed PMID: 19923337. Epub 2009/11/20.
76. Clark RA, Inglis SC, McAlister FA, Ball J, Lewinter C, Cullington D, et al. Remote (non-invasive) monitoring in heart failure: Effect on length of stay, quality of life, knowledge, adherance and satisfaction in 8,323 heart failure patients: A systematic review. European Heart Journal. 2010;31:944-5.
77. Clark RA, Inglis SC, McAlister FA, Ball J, Lewinter C, Cullington D, et al. Results from a systematic review and meta-analysis of remote (non-invasive) monitoring in 8,323 heart failure patients on length of stay, quality of life, knowledge, compliance and satisfaction. European Journal of Heart Failure, Supplement. 2010;9:S51-S2.
78. Inglis SC, Clark RA, McAlister FA, Ball J, Lewinter C, Cullington D, et al. Benefits of structured telephone support or telemonitoring in heart failure on mortality, hospitalisation and cost: A meta-analysis of 8,323 heart failure patients. European Heart Journal. 2010;31:878.
79. Inglis SC, Clark RA, McAlister FA, Ball J, Lewinter C, Cullington D, et al. A meta-analysis of 8,323 heart failure patients receiving structured telephone support or non-invasive telemonitoring to reduce mortality, hospitalisation and cost. European Journal of Heart Failure, Supplement. 2010;9:S179.
80. Polisena J, Tran K, Cimon K, Hutton B, McGill S, Palmer K, et al. Home telemonitoring for congestive heart failure: A systematic review and meta-analysis. Journal of Telemedicine and Telecare. 2010;16(2):68-76.
81. Birati EY, Roth A. Telecardiology. Israel Medical Association Journal. 2011;13(8):498-503.
82. Burri H, Heidbuchel H, Jung W, Brugada P. Remote monitoring: a cost or an investment? Europace. 2011 May;13 Suppl 2:ii44-8. PubMed PMID: 21518749. Epub 2011/04/29.
83. Kraai IH, Luttik ML, de Jong RM, Jaarsma T, Hillege HL. Heart failure patients monitored with telemedicine: patient satisfaction, a review of the literature. J Card Fail. 2011 Aug;17(8):684-90. PubMed PMID: 21807331. Epub 2011/08/03.
84. Gurne O, Conraads V, Missault L, Mullens W, Vachierys JL, Van Mieghem W, et al. A critical review on telemonitoring in heart failure. Acta Cardiol. 2012 Aug;67(4):439-44. PubMed PMID: 22997998. Epub 2012/09/25.
85. Radhakrishnan K, Jacelon C. Impact of telehealth on patient self-management of heart failure: a review of literature. J Cardiovasc Nurs. 2012 Jan-Feb;27(1):33-43. PubMed PMID: 21558862. Epub 2011/05/12.
86. Stoyanov N, Paul V. Clinical use of telemonitoring in chronic heart failure: keeping up with the times or misuse of time? Curr Heart Fail Rep. 2012 Mar;9(1):75-80. PubMed PMID: 22124933. Epub 2011/11/30.

87. Clark RA, Conway A, Inglis SC, Horton-Breshears M, Cleland JGF. Not all systematic reviews are systematic: A meta-review of the quality of current systematic reviews and meta-analyses for remote monitoring in heart failure. European Journal of Heart Failure. 2013;12:S229.
88. Pandor A. Home telemonitoring or structured telephone support programmes for patients with heart failure. Health Technology Assessment. 2013;17(32).
89. Pandor A, Gomersall T, Stevens JW, Wang J, Al-Mohammad A, Bakhai A, et al. Remote monitoring after recent hospital discharge in patients with heart failure: A systematic review and network meta-analysis. Heart. 2013;99(23):1717-26.

Chronic respiratory diseases

1. Curtin K, Hayes BD, Holland CL, Katz LA. Computer-generated intervention for asthma population care management. Effective clinical practice : ECP. 1998;1(1):43-6.
2. Atherton M. Outcome measures of efficacy associated with a web-enabled asthma self-management programme: Findings from a quasi-experiment. Disease Management and Health Outcomes. 2000;8(4):233-42.
3. Finkelstein J, O'Connor G, Friedmann RH. Development and implementation of the home asthma telemonitoring (HAT) system to facilitate asthma self-care. Stud Health Technol Inform. 2001;84(Pt 1):810-4. PubMed PMID: 11604847. Epub 2001/10/18.
4. Steel S, Lock S, Johnson N, Martinez Y, Marquilles E, Bayford R. A feasibility study of remote monitoring of asthmatic patients. J Telemed Telecare. 2002;8(5):290-6. PubMed PMID: 12396858. Epub 2002/10/25.
5. Chang BL, Omery A, Mayo A. Use of personal digital assistants by adolescents with severe asthma: can they enhance patient outcomes? AACN clinical issues. 2003 Aug;14(3):379-91; quiz 94-6. PubMed PMID: 12909806. Epub 2003/08/12.
6. Alonso A. A new model for home care for COPD. Studies in health technology and informatics. 2004;103:368-73.
7. Anhoj J, Moldrup C. Feasibility of collecting diary data from asthma patients through mobile phones and SMS (short message service): response rate analysis and focus group evaluation from a pilot study. J Med Internet Res. 2004 Dec 2;6(4):e42. PubMed PMID: 15631966. Pubmed Central PMCID: Pmc1550628. Epub 2005/01/06.
8. Magrabi F, Lovell NH, Henry RL, Celler BG. Designing home telecare: a case study in monitoring cystic fibrosis. Telemed J E Health. 2005 Dec;11(6):707-19. PubMed PMID: 16430391. Epub 2006/01/25.
9. Rasmussen LM, Phanareth K, Nolte H, Backer V. Internet-based monitoring of asthma: a long-term, randomized clinical study of 300 asthmatic subjects. The Journal of allergy and clinical immunology. 2005 Jun;115(6):1137-42. PubMed PMID: 15940125. Epub 2005/06/09.
10. Ryan D, Cobern W, Wheeler J, Price D, Tarassenko L. Mobile phone technology in the management of asthma. J Telemed Telecare. 2005;11 Suppl 1:43-6. PubMed PMID: 16035991. Epub 2005/07/23.
11. de Toledo P, Jimenez S, del Pozo F, Roca J, Alonso A, Hernandez C. Telemedicine experience for chronic care in COPD. IEEE transactions on information technology in biomedicine : a publication of the IEEE Engineering in Medicine and Biology Society. 2006;10(3):567-73.
12. Joseph CL, Peterson E, Havstad S, Johnson CC, Hoerauf S, Stringer S, et al. A web-based, tailored asthma management program for urban African-American high school

students. American journal of respiratory and critical care medicine. 2007;175(9):888-95. PubMed PMID: CN-00586819.
13. Pinnock H, Adlem L, Gaskin S, Harris J, Snellgrove C, Sheikh A. Accessibility, clinical effectiveness, and practice costs of providing a telephone option for routine asthma reviews: Phase IV controlled implementation study. British Journal of General Practice. 2007;57(542):714-22.
14. Whitten P, Mickus M. Home telecare for COPD/CHF patients: outcomes and perceptions. J Telemed Telecare. 2007;13(2):69-73. PubMed PMID: 17359569. Epub 2007/03/16.
15. Willems DCM, Joore MA, Hendriks JJE, Wouters EFM, Severens JL. Cost-effectiveness of a nurse-led telemonitoring intervention based on peak expiratory flow measurements in asthmatics: Results of a randomised controlled trial. Cost Effectiveness and Resource Allocation. 2007;5.
16. Donald KJ, McBurney H, Teichtahl H, Irving L, Browning C, Rubinfeld A, et al. Telephone based asthma management - financial and individual benefits. Australian family physician. 2008;37(4):272-5.
17. Liu WT, Wang CH, Lin HC, Lin SM, Lee KY, Lo YL, et al. Efficacy of a cell phone-based exercise programme for COPD. European Respiratory Journal. 2008;32(3):651-9.
18. Trappenburg JCA, Niesink A, De Weert-Van Oene GH, Van Der Zeijden H, Van Snippenburg R, Peters A, et al. Effects of telemonitoring in patients with chronic obstructive pulmonary disease. Telemedicine and e-Health. 2008;14(2):138-46.
19. Willems DCM, Joore MA, Hendriks JJE, Nieman FHM, Severens JL, Wouters EFM. The effectiveness of nurse-led telemonitoring of asthma: Results of a randomized controlled trial. Journal of Evaluation in Clinical Practice. 2008;14(4):600-9.
20. Feldstein A, Vollmer W, Rand C. Automated phone calls improved adherence to inhaled corticosteroids. Value in Health. 2009;12(7):A490.
21. Van Der Meer V, Bakker MJ, Van Den Hout WB, Rabe KF, Sterk PJ, Kievit J, et al. Internet-based self-management plus education compared with usual care in asthma: A randomized trial. Annals of Internal Medicine. 2009;151(2):110-20.
22. Vitacca M, Bianchi L, Guerra A, Fracchia C, Spanevello A, Balbi B, et al. Tele-assistance in chronic respiratory failure patients: a randomised clinical trial (Provisional abstract). European Respiratory Journal [Internet]. 2009; (2):[411-8 pp.]. Available from: http://www.ersj.org.uk/content/33/2/411.short.
23. Lewis KE, Annandale JA, Warm DL, Hurlin C, Lewis MJ, Lewis L. Home telemonitoring and quality of life in stable, optimised chronic obstructive pulmonary disease. Journal of Telemedicine and Telecare. 2010;16(5):253-9.
24. Lewis KE, Annandale JA, Warm DL, Rees SE, Hurlin C, Blyth H, et al. Does home telemonitoring after pulmonary rehabilitation reduce healthcare use in optimized COPD? A pilot randomized trial. Copd. 2010 Feb;7(1):44-50. PubMed PMID: 20214462. Epub 2010/03/11.
25. Wilson SR, Strub P, Buist AS, Knowles SB, Lavori PW, Lapidus J, et al. Shared treatment decision making improves adherence and outcomes in poorly controlled asthma. American Journal of Respiratory and Critical Care Medicine. 2010;181(6):566-77.
26. Cameron-Tucker H, Joseph L, Edwards B, Wood-Baker R. Telephone health-mentoring, a walking action plan and rehabilitation. Respirology. 2011;16:33.
27. Hashimoto S, Ten Brinke A, Roldaan AC, Van Veen IH, Moller GM, Sont JK, et al. Internet-based tapering of oral corticosteroids in severe asthma: A pragmatic randomised controlled trial. Thorax. 2011;66(6):514-20.

28. Liu WT, Huang CD, Wang CH, Lee KY, Lin SM, Kuo HP. A mobile telephone-based interactive self-care system improves asthma control. European Respiratory Journal. 2011;37(2):310-7.
29. Ruhle KH, Domanski U, Franke KJ, Bloch M, Nilius G. Telemedicine and training in COPD. Pneumologie. 2011;65(10):596-601.
30. Sicotte C, Pare G, Morin S, Potvin J, Moreault MP. Effects of home telemonitoring to support improved care for chronic obstructive pulmonary diseases. Telemedicine journal and e-health : the official journal of the American Telemedicine Association. 2011;17(2):95-103.
31. Stickland MK, Jourdain T, Wong EYL, Rodgers WM, Jendzjowsky NG, MacDonald GF. Using Telehealth technology to deliver pulmonary rehabilitation to patients with chronic obstructive pulmonary disease. Canadian Respiratory Journal. 2011;18(4):216-20.
32. van der Meer V, van den Hout WB, Bakker MJ, Rabe KF, Sterk PJ, Assendelft WJJ, et al. Cost-effectiveness of internet-based self-management compared with usual care in Asthma. PLoS ONE. 2011;6(11).
33. Antoniades NC, Rochford PD, Pretto JJ, Pierce RJ, Gogler J, Steinkrug J, et al. Pilot study of remote telemonitoring in COPD. Telemedicine journal and e-health : the official journal of the American Telemedicine Association. 2012;18(8):634-40.
34. Araújo L, Jacinto T, Moreira A, Castel-Branco MG, Delgado L, Costa-Pereira A, et al. Clinical efficacy of web-based versus standard asthma self-management. Journal of investigational allergology & clinical immunology. 2012;22(1):28-34. PubMed PMID: CN-00834039.
35. Bischoff EWMA, Akkermans R, Bourbeau J, Van Weel C, Vercoulen JH, Schermer TRJ. Comprehensive self management and routine monitoring in chronic obstructive pulmonary disease patients in general practice: Randomised controlled trial. BMJ (Online). 2012;345(7885).
36. Chau JPC, Lee DTF, Yu DSF, Chow AYM, Yu WC, Chair SY, et al. A feasibility study to investigate the acceptability and potential effectiveness of a telecare service for older people with chronic obstructive pulmonary disease. International Journal of Medical Informatics. 2012;81(10):674-82.
37. Lv Y, Zhao H, Liang Z, Dong H, Liu L, Zhang D, et al. A mobile phone short message service improves perceived control of asthma: a randomized controlled trial. Telemedicine journal and e-health : the official journal of the American Telemedicine Association. 2012;18(6):420-6.
38. Murgia F, Cotognini C, Montemitro E, Cilli M, Renzetti E, Lucidi V, et al. Evaluation of compliance to telehomecare (THC) in a group of patients with cystic fibrosis (CF) in a period of 2 years. Clin Ter. 2012;163(3):e111-4. PubMed PMID: 22964701. Epub 2012/09/12.
39. Nield M, Hoo GWS. Real-time telehealth for COPD self-management using skype. COPD: Journal of Chronic Obstructive Pulmonary Disease. 2012;9(6):611-9.
40. Pinnock H, McCloughlan L, Todd A, Hanley J, Lewis S, Krishan A, et al. Clinical effectiveness of telemonitoring for chronic obstructive pulmonary disease (COPD): Randomised controlled trial. Thorax. 2012;67:A27.
41. Rikkers-Mutsaerts ERVM, Winters AE, Bakker MJ, Van Stel HF, Van Der Meer V, De Jongste JC, et al. Internet-based self-management compared with usual care in adolescents with asthma: A randomized controlled trial. Pediatric Pulmonology. 2012;47(12):1170-9.

42. Roberts A, Garrett L, Godden DJ. Can telehealth deliver for rural Scotland? Lessons from the Argyll & Bute Telehealth Programme. Scott Med J. 2012 Feb;57(1):33-7. PubMed PMID: 22408213. Epub 2012/03/13.
43. Ryan D, Price D, Musgrave SD, Malhotra S, Lee AJ, Ayansina D, et al. Clinical and cost effectiveness of mobile phone supported self monitoring of asthma: multicentre randomised controlled trial. BMJ (Clinical research ed). 2012;344.
44. Van Gaalen JL, Hashimoto S, Sont JK. Telemanagement in asthma: an innovative and effective approach. Curr Opin Allergy Clin Immunol. 2012 Jun;12(3):235-40. PubMed PMID: 22475997. Epub 2012/04/06.
45. Venter A, Burns R, Hefford M, Ehrenberg N. Results of a telehealth-enabled chronic care management service to support people with long-term conditions at home. Journal of Telemedicine and Telecare. 2012;18(3):172-5.
46. Baptist AP, Ross JA, Yang Y, Song PXK, Clark NM. A randomized controlled trial of a self-regulation intervention for older adults with asthma. Journal of the American Geriatrics Society. 2013;61(5):747-53.
47. Burns P, Jones SC, Iverson D, Caputi P. AsthmaWise-a field of dreams? the results of an online education program targeting older adults with asthma. Journal of Asthma. 2013;50(7):737-44.
48. Holland A. Telehealth reduces hospital admission rates in patients with COPD. Journal of physiotherapy. 2013 Jun;59(2):129. PubMed PMID: 23663801. Epub 2013/05/15.
49. Holland AE, Hill CJ, Rochford P, Fiore J, Berlowitz DJ, McDonald CF. Telerehabilitation for people with chronic obstructive pulmonary disease: feasibility of a simple, real time model of supervised exercise training. J Telemed Telecare. 2013 Jun;19(4):222-6. PubMed PMID: 23666438. Epub 2013/05/15.
50. Jodar-Sanchez F, Ortega F, Parra C, Gomez-Suarez C, Jordan A, Perez P, et al. Implementation of a telehealth programme for patients with severe chronic obstructive pulmonary disease treated with long-term oxygen therapy. J Telemed Telecare. 2013 Jan;19(1):11-7. PubMed PMID: 23393057. Epub 2013/02/09.
51. Licskai CJ, Sands TW, Ferrone M. Development and pilot testing of a mobile health solution for asthma self-management: Asthma action plan smartphone application pilot study. Canadian Respiratory Journal. 2013;20(4):301-6.
52. Liu SX, Lee MC, Atakhorrami M, Tatousek J, McCormack M, Yung R, et al. Economic assessment of home-based COPD management programs. COPD: Journal of Chronic Obstructive Pulmonary Disease. 2013;10(6):640-9.
53. Pedone C, Chiurco D, Scarlata S, Incalzi RA. Efficacy of multiparametric telemonitoring on respiratory outcomes in elderly people with COPD: a randomized controlled trial. BMC Health Serv Res. 2013;13:82. PubMed PMID: 23497109. Pubmed Central PMCID: Pmc3680224. Epub 2013/03/19.
54. Pinnock H, Hanley J, McCloughan L, Todd A, Krishan A, Lewis S, et al. Effectiveness of telemonitoring integrated into existing clinical services on hospital admission for exacerbation of chronic obstructive pulmonary disease: Researcher blind, multicentre, randomised controlled trial. BMJ (Online). 2013;347.
55. Schou L, Ostergaard B, Rydahl-Hansen S, Rasmussen LS, Emme C, Jakobsen AS, et al. A randomised trial of telemedicine-based treatment versus conventional hospitalisation in patients with severe COPD and exacerbation - effect on self-reported outcome. J Telemed Telecare. 2013 Apr 23. PubMed PMID: 23612519. Epub 2013/04/25.

56. Sorknaes AD, Bech M, Madsen H, Titlestad IL, Hounsgaard L, Hansen-Nord M, et al. The effect of real-time teleconsultations between hospital-based nurses and patients with severe COPD discharged after an exacerbation. J Telemed Telecare. 2013;19(8):466-74. PubMed PMID: 24227799. Epub 2013/11/15.
57. van Gaalen JL, Beerthuizen T, van der Meer V, van Reisen P, Redelijkheid GW, Snoeck-Stroband JB, et al. Long-term outcomes of internet-based self-management support in adults with asthma: randomized controlled trial. J Med Internet Res. 2013;15(9):e188. PubMed PMID: 24028826. Pubmed Central PMCID: Pmc3785973. Epub 2013/09/14. eng.
58. Van Sickle D, Magzamen S, Truelove S, Morrison T. Remote monitoring of inhaled bronchodilator use and weekly feedback about asthma management: an open-group, short-term pilot study of the impact on asthma control. PLoS One. 2013;8(2):e55335. PubMed PMID: 23460785. Pubmed Central PMCID: Pmc3584103. Epub 2013/03/06.
59. Walters J, Cameron-Tucker H, Wills K, Schuz N, Scott J, Robinson A, et al. Effects of telephone health mentoring in community-recruited chronic obstructive pulmonary disease on self-management capacity, quality of life and psychological morbidity: A randomised controlled trial. BMJ Open. 2013;3(9).
60. Walters JAE, Wills K, Schuez N, Cameron-Tucker H, Courtney-Pratt H, Nelson M, et al. Telephone health mentoring improves selfmanagement capacity in community-recruited copd. Respirology. 2013;18:50.
61. Jaana M, Pare G, Sicotte C. Home telemonitoring for respiratory conditions: A systematic review. American Journal of Managed Care. 2009;15(5):313-20.
62. McLean S, Liu J, Pagliari C, Car J, Sheikh A. Telehealthcare for asthma. Cochrane Database of Systematic Reviews. 2009 (2).
63. McLean S, Liu J, Pagliari C, Car J, Sheikh A. Telehealthcare for chronic obstructive pulmonary disease. Cochrane Database of Systematic Reviews. 2009 (2).
64. McLean S, Chandler D, Nurmatov U, Liu J, Pagliari C, Car J, et al. Telehealthcare for asthma. Cochrane Database Syst Rev. 2010 (10):Cd007717. PubMed PMID: 20927763. Epub 2010/10/12.
65. Marcano Belisario JS, Huckvale K, Greenfield G, Car J, Gunn LH. Smartphone and tablet self management apps for asthma. Cochrane Database Syst Rev. 2013;11:Cd010013. PubMed PMID: 24282112. Epub 2013/11/28.
66. Wainwright C, Wootton R. A review of telemedicine and asthma. Disease Management and Health Outcomes. 2003;11(9):557-63.
67. Bussey-Smith KL, Rossen RD. A systematic review of randomized control trials evaluating the effectiveness of interactive computerized asthma patient education programs. Annals of Allergy, Asthma and Immunology. 2007;98(6):507-16.
68. Duvvuri VRSK, Jianhong W. Information and communication technology developments in asthma management: A systematic review. Indian Journal of Medical Sciences. 2007;61(4):221-41.
69. Bartoli L, Zanaboni P, Masella C, Ursini N. Systematic review of telemedicine services for patients affected by chronic obstructive pulmonary disease (COPD). Telemed J E Health. 2009 Nov;15(9):877-83. PubMed PMID: 19919194. Epub 2009/11/19.
70. Polisena J, Tran K, Cimon K, Hutton B, McGill S, Palmer K, et al. Home telehealth for chronic obstructive pulmonary disease: a systematic review and meta-analysis. J Telemed Telecare. 2010;16(3):120-7. PubMed PMID: 20197355. Epub 2010/03/04.

71. Bolton CE, Waters CS, Peirce S, Elwyn G. Insufficient evidence of benefit: A systematic review of home telemonitoring for COPD. Journal of Evaluation in Clinical Practice. 2011;17(6):1216-22.
72. Cox NS, Alison JA, Rasekaba T, Holland AE. Telehealth in cystic fibrosis: a systematic review. J Telemed Telecare. 2012 Mar;18(2):72-8. PubMed PMID: 22198961. Epub 2011/12/27.
73. Franek J. Home telehealth for patients with chronic obstructive pulmonary disease (COPD): An evidence-based analysis. Ontario Health Technology Assessment Series. 2012;12(11):1-58.

Cancer

1. Doolittle GC, Harmon A, Williams A, Allen A, Boysen CD, Wittman C, et al. A cost analysis of a tele-oncology practice. Journal of telemedicine and telecare. 1997;3 Suppl 1:20-2.
2. Doolittle GC, Williams A, Harmon A, Allen A, Boysen CD, Wittman C, et al. A cost measurement study for a tele-oncology practice. Journal of telemedicine and telecare. 1998;4(2):84-8.
3. Kunkler IH, Rafferty P, Hill D, Henry M, Foreman D. A pilot study of tele-oncology in Scotland. Journal of telemedicine and telecare. 1998;4(2):113-9.
4. Jones R, Pearson J, McGregor S, Cawsey AJ, Barrett A, Craig N, et al. Randomised trial of personalised computer based information for cancer patients. Bmj. 1999 Nov 6;319(7219):1241-7. PubMed PMID: 10550090. Pubmed Central PMCID: Pmc28275. Epub 1999/11/05.
5. Sandgren AK, McCaul KD, King B, O'Donnell S, Foreman G. Telephone therapy for patients with breast cancer. Oncology nursing forum. 2000;27(4):683-8.
6. Sezeur A, Degramont A, Touboul E, Mosnier H. Teleconsultation before chemotherapy for recently operated on patients. Am J Surg. 2001 Jul;182(1):49-51. PubMed PMID: 11532415. Epub 2001/09/05.
7. Stalfors J, Edstrom S, Bjork-Eriksson T, Mercke C, Nyman J, Westin T. Accuracy of tele-oncology compared with face-to-face consultation in head and neck cancer case conferences. J Telemed Telecare. 2001;7(6):338-43. PubMed PMID: 11747635. Epub 2001/12/19.
8. Williams JG, Cheung WY, Chetwynd N, Cohen DR, El-Sharkawi S, Finlay I, et al. Pragmatic randomised trial to evaluate the use of patient held records for the continuing care of patients with cancer. Quality in Health Care. 2001;10(3):159-65.
9. Lecouturier J, Crack L, Mannix K, Hall RH, Bond S. Evaluation of a patient-held record for patients with cancer. Eur J Cancer Care (Engl). 2002 Jun;11(2):114-21. PubMed PMID: 12099947. Epub 2002/07/09.
10. Lieberman MA, Golant M, Giese-Davis J, Winzlenberg A, Benjamin H, Humphreys K, et al. Electronic support groups for breast carcinoma: A clinical trial of effectiveness. Cancer. 2003;97(4):920-5.
11. Bielli E, Carminati F, La Capra S, Lina M, Brunelli C, Tamburini M. A Wireless Health Outcomes Monitoring System (WHOMS): Development and field testing with cancer patients using mobile phones. BMC Medical Informatics and Decision Making. 2004;4.

12. Doolittle GC, Williams AR, Spaulding A, Spaulding RJ, Cook DJ. A cost analysis of a tele-oncology practice in the United States. Journal of Telemedicine and Telecare. 2004;10(SUPPL. 1):27-9.
13. Lieberman MA, Goldstein BA. Self-help on-line: an outcome evaluation of breast cancer bulletin boards. Journal of health psychology. 2005 Nov;10(6):855-62. PubMed PMID: 16176962. Epub 2005/09/24.
14. Owen JE, Klapow JC, Roth DL, Shuster Jr JL, Bellis J, Meredith R, et al. Randomized pilot of a self-guided Internet coping group for women with early-stage breast cancer. Annals of Behavioral Medicine. 2005;30(1):54-64.
15. Basch E, Artz D, Iasonos A, Speakman J, Shannon K, Lin K, et al. Evaluation of an online platform for cancer patient self-reporting of chemotherapy toxicities. J Am Med Inform Assoc. 2007 May-Jun;14(3):264-8. PubMed PMID: 17329732. Pubmed Central PMCID: Pmc2244885. Epub 2007/03/03.
16. Brink JL, Moorman PW, Boer MF, Hop WC, Pruyn JF, Verwoerd CD, et al. Impact on quality of life of a telemedicine system supporting head and neck cancer patients: a controlled trial during the postoperative period at home. Journal of the American Medical Informatics Association : JAMIA. 2007;14(2):198-205. PubMed PMID: CN-00577634.
17. Chumbler NR, Mkanta WN, Richardson LC, Harris L, Darkins A, Kobb R, et al. Remote patient-provider communication and quality of life: empirical test of a dialogic model of cancer care. Journal of telemedicine and telecare. 2007;13(1):20-5.
18. Kim AS, Lee ES, Kim SH. Effects of telephone intervention as supportive nursing on self-care practices and qualify of life for gynecological cancer patients under chemotherapy. Taehan Kanho Hakhoe chi. 2007;37(5):744-53.
19. Kunkler IH, Prescott RJ, Lee RJ, Brebner JA, Cairns JA, Fielding RG, et al. TELEMAM: A cluster randomised trial to assess the use of telemedicine in multi-disciplinary breast cancer decision making. European Journal of Cancer. 2007;43(17):2506-14.
20. Matthew AG, Currie KL, Irvine J, Ritvo P, Santa Mina D, Jamnicky L, et al. Serial personal digital assistant data capture of health-related quality of life: A randomized controlled trial in a prostate cancer clinic. Health and Quality of Life Outcomes. 2007;5.
21. Matthew AG, Currie KL, Ritvo P, Nam R, Nesbitt ME, Kalnin RW, et al. Personal digital assistant data capture: The future of quality of life measurement in prostate cancer treatment. Journal of Oncology Practice. 2007;3(3):115-20.
22. Sandgren AK, McCaul KD. Long-term telephone therapy outcomes for breast cancer patients. Psychooncology. 2007 Jan;16(1):38-47. PubMed PMID: 16862634. Epub 2006/07/25.
23. van den Brink JL, Moorman PW, de Boer MF, Hop WCJ, Pruyn JFA, Verwoerd CDA, et al. Impact on Quality of Life of a Telemedicine System Supporting Head and Neck Cancer Patients: A Controlled Trial During the Postoperative Period at Home. Journal of the American Medical Informatics Association. 2007;14(2):198-205.
24. Gustafson DH, Hawkins R, McTavish F, Pingree S, Chen WC, Volrathongchai K, et al. Internet-Based Interactive Support for Cancer Patients: Are Integrated Systems Better? The Journal of communication. 2008 Jun;58(2):238-57. PubMed PMID: 21804645. Pubmed Central PMCID: Pmc3144782. Epub 2008/06/01.
25. Montgomery DA, Krupa K, Wilson C, Cooke TG. Automated telephone follow-up after breast cancer: An acceptability and feasibility pilot study. British Journal of Cancer. 2008;99(5):704-10.

26. Wise M, Han JY, Shaw B, McTavish F, Gustafson DH. Effects of using online narrative and didactic information on healthcare participation for breast cancer patients. Patient Education and Counseling. 2008;70(3):348-56.
27. Beaver K, Tysver-Robinson D, Campbell M, Twomey M, Williamson S, Hindley A, et al. Comparing hospital and telephone follow-up after treatment for breast cancer: randomised equivalence trial. Bmj. 2009;338:a3147. PubMed PMID: 19147478. Pubmed Central PMCID: Pmc2628299. Epub 2009/01/17.
28. Head B. Telehealth symptom management in head and neck cancer. Psycho-Oncology. 2009;18:S45.
29. Kearney N, McCann L, Norrie J, Taylor L, Gray P, McGee-Lennon M, et al. Evaluation of a mobile phone-based, advanced symptom management system (ASyMS) in the management of chemotherapy-related toxicity. Supportive care in cancer : official journal of the Multinational Association of Supportive Care in Cancer. 2009 Apr;17(4):437-44. PubMed PMID: 18953579. Epub 2008/10/28.
30. Khayat M, Head BA, Studts JL, Keeney CA, Bumpous J, Pfeifer MP. Feasibility and acceptance of a telehealth intervention during initial treatment for head and neck cancer. Journal of Supportive Oncology. 2009;7(5):206.
31. Pare G, Sicotte C, Chekli M, Jaana M, De Blois C, Bouchard M. A pre-post evaluation of a telehomecare program in oncology and palliative care. Telemedicine and e-Health. 2009;15(2):154-9.
32. Salonen P, Tarkka MT, Kellokumpu-Lehtinen PL, Astedt-Kurki P, Luukkaala T, Kaunonen M. Telephone intervention and quality of life in patients with breast cancer. Cancer Nursing. 2009;32(3):177-90.
33. Watson M, White C, Ashley S, Bryan S, Davolls S, Hopwood P. Problem focussed interactive telephone therapy - ProFITT(copyright): Results of a phase 2 trial. Psycho-Oncology. 2009;18:S64-S5.
34. Diefenbach M. Choosing prostate cancer therapy with multimedia software. Community Oncology. 2010;7(8):359-60.
35. Hawkes A, Pakenham K, Courneya K, Peter B, Chambers S. 'Canchange': A trial of a telephone-delivered lifestyle intervention for colorectal cancer (CRC) survivors. Asia-Pacific Journal of Clinical Oncology. 2010;6:193.
36. Keeney CE, Head BA, Myers J, Appana SN, Pfeifer MP. The impact of a telehealth intervention on symptom burden and quality of life for head and neck cancer patients. Journal of Supportive Oncology. 2010;8(5):A10-A1.
37. Kroenke K, Theobald D, Wu J, Norton K, Morrison G, Carpenter J, et al. Effect of telecare management on pain and depression in patients with cancer: A randomized trial. JAMA - Journal of the American Medical Association. 2010;304(2):163-71.
38. Ruland CM, Holte HH, Røislien J, Heaven C, Hamilton GA, Kristiansen J, et al. Effects of a computer-supported interactive tailored patient assessment tool on patient care, symptom distress, and patients' need for symptom management support: a randomized clinical trial. Journal of the American Medical Informatics Association : JAMIA. 2010;17(4):403-10. PubMed PMID: CN-00762565.
39. Thaker DA, Sabesan S. Cost-effectiveness analysis of videolinked medical oncology outreach clinics: A townsville experience. Asia-Pacific Journal of Clinical Oncology. 2010;6:140.
40. Cox A, Illsley M, Knibb W, Lucas C, O'Driscoll M, Potter C, et al. The acceptability of e-technology to monitor and assess patient symptoms following palliative radiotherapy for lung cancer. Palliat Med. 2011 Oct;25(7):675-81. PubMed PMID: 21474620. Epub 2011/04/09.

41. Hawkes A, Pakenham K, Courneya KS, Patrao T. A randomised controlled trial of the effects of a telephone-delivered program on health behaviours and quality of life for colorectal cancer survivors ('canchange'). Asia-Pacific Journal of Clinical Oncology. 2011;7:80-1.
42. Hawkins RP, Pingree S, Baker TB, Roberts LJ, Shaw BR, McDowell H, et al. Integrating eHealth with human services for breast cancer patients. Translational Behavioral Medicine. 2011;1(1):146-54.
43. Head BA, Keeney C, Studts JL, Khayat M, Bumpous J, Pfeifer M. Feasibility and Acceptance of a Telehealth Intervention to Promote Symptom Management during Treatment for Head and Neck Cancer. Journal of Supportive Oncology. 2011;9(1):e1-e11.
44. Izquierdo F, Gracia J, Guerra M, Blasco JA, Andradas E. Health technology assessment-based development of a Spanish breast cancer patient decision aid. Int J Technol Assess Health Care. 2011 Oct;27(4):363-8. PubMed PMID: 22004778. Epub 2011/10/19.
45. Kimman ML, Dirksen CD, Voogd AC, Falger P, Gijsen BCM, Thuring M, et al. Nurse-led telephone follow-up and an educational group programme after breast cancer treatment: Results of a 2 x 2 randomised controlled trial. European Journal of Cancer. 2011;47(7):1027-36.
46. Seckin G. Informational and decisional empowerment in online health support communities: Initial psychometric validation of the Cyber Info-Decisional Empowerment Scale (CIDES) and preliminary data from administration of the scale. Supportive Care in Cancer. 2011;19(12):2057-61.
47. Thaker DA, Sabesan SS, Van Houts B, Bryett A, Olver IN. Tele oncology clinics in rural australia: A cost-effective cancer care model. European Journal of Cancer. 2011;47:S213-S4.
48. Wagner LI, Duffecy J, Lehman KA, Sanford SD, Begale M, Nawacki E, et al. Randomized clinical trial to evaluate an e-health intervention for fear of cancer recurrence, anxiety, and depression among cancer survivors. Journal of Clinical Oncology. 2011;29(15).
49. Bartlett YK, Selby DL, Newsham A, Keding A, Forman D, Brown J, et al. Developing a useful, user-friendly website for cancer patient follow-up: Users' perspectives on ease of access and usefulness. European Journal of Cancer Care. 2012;21(6):747-57.
50. Berry DL, Hong F, Halpenny B, Wang Q, Partridge AH, Fann JR, et al. Electronic self-report assessment for cancer: Results of a multisite randomized trial. Journal of Clinical Oncology. 2012;30(15).
51. Ligibel JA, Meyerhardt J, Pierce JP, Najita J, Shockro L, Campbell N, et al. Impact of a telephone-based physical activity intervention upon exercise behaviors and fitness in cancer survivors enrolled in a cooperative group setting. Breast Cancer Research and Treatment. 2012;132(1):205-13.
52. Sabesan S, Larkins S, Evans R, Varma S, Andrews A, Beuttner P, et al. Telemedicine for rural cancer care in North Queensland: bringing cancer care home. The Australian journal of rural health. 2012 Oct;20(5):259-64. PubMed PMID: 22998200. Epub 2012/09/25. eng.
53. Sugawara Y, Narimatsu H, Hozawa A, Shao L, Otani K, Fukao A. Cancer patients on Twitter: a novel patient community on social media. BMC research notes. 2012;5:699.
54. Badger T, Segrin C, Hepworth J, Pasvogel A, Lopez AM. Counseling and health education delivered by telephone and skype to improve quality of life for breast cancer survivors and supportive partners. Psycho-Oncology. 2013;22:143-4.

55. Beatty L, Koczwara B, Wade T. Cancer coping online: Findings and lessons learned from a phase II RCT of an ehealth program for reducing cancer-distress. Asia-Pacific Journal of Clinical Oncology. 2013;9:120.
56. Duffecy J, Sanford S, Wagner L, Begale M, Nawacki E, Mohr DC. Project onward: an innovative e-health intervention for cancer survivors. Psychooncology. 2013 Apr;22(4):947-51. PubMed PMID: 22438297. Pubmed Central PMCID: Pmc3387296. Epub 2012/03/23.
57. Geller M, Petzel S, Vogel R, McClellan M, Jacko J, Cragg J, et al. An interactive website for patients with ovarian cancer and their care givers-can we improve quality of life? Gynecologic Oncology. 2013;130(1):e145.
58. Osei DK, Lee JW, Modest NN, Pothier PK. Effects of an online support group for prostate cancer survivors: a randomized trial. Urologic nursing. 2013;33(3):123-33.
59. Petzel S, Vogel RI, Chan D, McClellan M, Gerber M, Cragg J, et al. Patient-centered ovarian cancer care: An interactive website to promote emotional quality of life for women and their caregivers. Psycho-Oncology. 2013;22:131.
60. Ruland CM, Maffei RM, Borosund E, Krahn A, Andersen T, Grimsbo GH. Evaluation of different features of an eHealth application for personalized illness management support: Cancer patients' use and appraisal of usefulness. International Journal of Medical Informatics. 2013;82(7):593-603.
61. Ventura F, Sawatzky R, Ohlen J, Karlsson P, Koinberg I. Evaluation of a web-based educational program for women diagnosed with breast cancer: why is the intervention effect absent? Stud Health Technol Inform. 2013;192:1132. PubMed PMID: 23920906. Epub 2013/08/08.
62. Wengstrom Y, Langius Eklof A, Sundberg K, Blomberg K. Symptom management for cancer patients via mobile phones. European Journal of Cancer. 2013;49:S62-S3.
63. Yount SE, Rothrock N, Bass M, Beaumont JL, Pach D, Lad T, et al. A Randomized Trial of Weekly Symptom Telemonitoring in Advanced Lung Cancer. Journal of Pain and Symptom Management. 2013.
64. Harrison JD, Young JM, Butow PN, Jorgensen M, Solomon MJ. Nurse-delivered telephone supportive care interventions: A systematic review. Asia-Pacific Journal of Clinical Oncology. 2010;6:238.
65. Aggarwal S. Electronic patient reported outcomes and data tool for chronic disease management (PROCDIM): Case in point prostate cancer. Value in Health. 2012;15(4):A201.
66. Kuijpers W, Groen WG, Aaronson NK, van Harten WH. A systematic review of web-based interventions for patient empowerment and physical activity in chronic diseases: relevance for cancer survivors. Journal of medical Internet research. 2013;15(2):e37.
67. Kuijpers W, Groen WG, Oldenburg HSA, Wouters MWJM, Aaronson NK, Van Harten WH. An interactive web portal for patient empowerment in cancer survivorship. European Journal of Cancer. 2013;49:S255-S6.

Stroke

1. Laver Kate E, George S, Thomas S, Deutsch Judith E, Crotty M. Virtual reality for stroke rehabilitation. Cochrane Database of Systematic Reviews [Internet]. 2011; (9).

Available from:
http://onlinelibrary.wiley.com/doi/10.1002/14651858.CD008349.pub2/abstract.
2. Sairanen T, Tatlisumak T. Finnish telestroke: An overview. European Research in Telemedicine. 2012;1(3-4):115-7.
3. Joubert J, Christie A, Laing J, Wilks B, Barnes I, De Bustos Medeiros E, et al. Telestroke: Long-term risk factor management - Part II. European Research in Telemedicine. 2013;2(2):57-67.
4. Grant JS, Elliott TR, Weaver M, Bartolucci AA, Giger JN. Telephone intervention with family caregivers of stroke survivors after rehabilitation. Stroke. 2002 Aug;33(8):2060-5. PubMed PMID: 12154263. Epub 2002/08/03.
5. Lai JC, Woo J, Hui E, Chan WM. Telerehabilitation - a new model for community-based stroke rehabilitation. J Telemed Telecare. 2004;10(4):199-205. PubMed PMID: 15273029. Epub 2004/07/27.
6. Piron L, Tonin P, Trivello E, Battistin L, Dam M. Motor tele-rehabilitation in post-stroke patients. Medical informatics and the Internet in medicine. 2004 Jun;29(2):119-25. PubMed PMID: 15370992. Epub 2004/09/17.
7. Lam YS, Man DW, Tam SF, Weiss PL. Virtual reality training for stroke rehabilitation. NeuroRehabilitation. 2006;21(3):245-53. PubMed PMID: CN-00577297.
8. Broeren J, Claesson L, Goude D, Rydmark M, Sunnerhagen KS. Virtual rehabilitation in an activity centre for community-dwelling persons with stroke. The possibilities of 3-dimensional computer games. Cerebrovascular diseases (Basel, Switzerland). 2008;26(3):289-96. PubMed PMID: CN-00669247.
9. Hornby TG, Campbell DD, Kahn JH, Demott T, Moore JL, Roth HR. Enhanced gait-related improvements after therapist- versus robotic-assisted locomotor training in subjects with chronic stroke: a randomized controlled study. Stroke; a journal of cerebral circulation. 2008;39(6):1786-92. PubMed PMID: CN-00639515.
10. LaMonte MP, Bahouth MN, Xiao Y, Hu P, Baquet CR, Mackenzie CF. Outcomes from a comprehensive stroke telemedicine program. Telemed J E Health. 2008 May;14(4):339-44. PubMed PMID: 18570562. Epub 2008/06/24.
11. Piron L, Turolla A, Tonin P, Piccione F, Lain L, Dam M. Satisfaction with care in post-stroke patients undergoing a telerehabilitation programme at home. J Telemed Telecare. 2008;14(5):257-60. PubMed PMID: 18633001. Epub 2008/07/18.
12. Joubert J, Joubert LB, Medeiros De Bustos E, Ware D, Jackson D, Harrison T, et al. Telestroke in stroke survivors. Cerebrovascular Diseases. 2009;27(SUPPL. 4):28-35.
13. Piron L, Turolla A, Agostini M, Zucconi C, Cortese F, Zampolini M, et al. Exercises for paretic upper limb after stroke: a combined virtual-reality and telemedicine approach. J Rehabil Med. 2009 Nov;41(12):1016-102. PubMed PMID: 19841835. Epub 2009/10/21. eng.
14. Taylor DM, Cameron JI, Walsh L, McEwen S, Kagan A, Streiner DL, et al. Exploring the feasibility of videoconference delivery of a self-management program to rural participants with stroke. Telemedicine and e-Health. 2009;15(7):646-54.
15. Piron L, Turolla A, Agostini M, Zucconi CS, Ventura L, Tonin P, et al. Motor learning principles for rehabilitation: a pilot randomized controlled study in poststroke patients. Neurorehabilitation and neural repair. 2010;24(6):501-8. PubMed PMID: CN-00767814.
16. Kuo YH, Chien YK, Wang WR, Chen CH, Chen LS, Liu CK. Development of a home-based telehealthcare model for improving the effectiveness of the chronic care of stroke patients. Kaohsiung Journal of Medical Sciences. 2012;28(1):38-43.

17. Ortmann S, Langendorfer P, Lanyi CS. Telemedical assistance for ambulant rehabilitation of stroke patients. Brain Injury. 2012;26(4-5):644-5.
18. Muir A, Wilson A, James K. Using telehealth to overcome barriers to access communication therapy: A case study of a successful service delivery model for an Aboriginal man with chronic communication impairment following stroke. International Journal of Stroke. 2013;8:23.
